# Data‐driven multi‐objective optimization via grid compatible simplex technique and desirability approach for challenging high throughput chromatography applications

**DOI:** 10.1002/btpr.2673

**Published:** 2018-10-09

**Authors:** Spyridon Konstantinidis, John P. Welsh, Nigel J. Titchener‐Hooker, David J. Roush, Ajoy Velayudhan

**Affiliations:** ^1^ Dept. of Biochemical Engineering The Advanced Centre for Biochemical Engineering, University College London London U.K.; ^2^ Biologics Process Research and Development, Merck & Co., Inc. Kenilworth, NJ USA

**Keywords:** chromatography, design of experiments, desirability, high throughput bioprocess development, multi‐objective optimization, Pareto front, Simplex optimization

## Abstract

Recently, a grid compatible Simplex variant has been demonstrated to identify optima consistently and rapidly in challenging high throughput (HT) applications in early bioprocess development. Here, this method is extended by deploying it to multi‐objective optimization problems. Three HT chromatography case studies are presented, each posing challenging early development situations and including three responses which were amalgamated by the adoption of the desirability approach. The suitability of a design of experiments (DoE) methodology per case study, using regression analysis in addition to the desirability approach, was evaluated for a large number of weights and in the presence of stringent and lenient performance requirements. Despite the adoption of high‐order models, this approach had low success in identification of the optimal conditions. For the deployment of the Simplex approach, the deterministic specification of the weights of the merged responses was avoided by including them as inputs in the formulated multi‐objective optimization problem, facilitating this way the decision making process. This, and the ability of the Simplex method to locate optima, rendered the presented approach highly successful in delivering rapidly operating conditions, which belonged to the Pareto set and offered a superior and balanced performance across all outputs compared to alternatives. Moreover, its performance was relatively independent of the starting conditions and required sub‐minute computations despite its higher order mathematical functionality compared to DoE techniques. These evidences support the suitability of the grid compatible Simplex method for early bioprocess development studies involving complex data trends over multiple responses. © 2018 The Authors Biotechnology Progress published by Wiley Periodicals, Inc. on behalf of American Institute of Chemical Engineers *Biotechnol. Prog*., 34:1393–1406, 2018

## Introduction

The elucidation and definition of the design space of a process is often the culmination of development efforts including the completion of experiments across multiple scales. Approximations of the design space can be obtained from the early stages of bioprocess development which will evolve, along with the process itself, until an optimized process has been established. In such early stages, high throughput (HT) studies are routinely implemented to identify attractive process conditions for further investigation which will aim to characterize them in detail. These studies are often facilitated by robotic automation and are frequently evaluated, for both upstream and downstream operations, with the adoption of model‐driven analyses such as design of experiments (DoE) (e.g., Refs. 1, 2, 3, 4). Multivariate data analysis is also emerging as a tool for multivariate statistical process control and for deconvoluting chromatograms in downstream processing.^5^ Here, regression analysis is used in a DoE approach to scout combinations of inputs so as to determine how they affect a set of objective functions, or outputs/responses. These can include quantities such as yield, impurity, and contaminant levels, and so on. In these investigations, a multi‐objective optimization problem can be defined and be addressed by overlaying the response surfaces of the considered outputs so as to obtain windows of operation in a graphical fashion.^6^

While such an approach for DoE‐based studies is straightforward for a small number of responses, it becomes increasingly complex as their number increases since numerous slices in both input and output multidimensional spaces need to be observed so as to determine the presence of an optimum across all responses and the holistic behavior of a process in its proximity. Hence, alternative techniques are used for such studies which seek to amalgamate even a large number of individual responses into a single objective or response.^6^ The graphical analysis of such a composite response is then simplified significantly as only slices in multidimensional input spaces need to be considered. In this multi‐objective optimization situation, such amalgamation techniques deliver scalar optima based on user defined criteria. The latter are often represented by weights on the considered responses whose definition poses challenges as it requires expert knowledge on a system which may not always be available.^7^ In multicriteria decision making applications, this challenge can be met by adopting a stochastic approach to deliver valuable information on the impact of the weights to the decision‐making process (e.g., Refs. 8, 9). Similar to this approach is the inclusion of the weights as inputs in the formulation of numerical multi‐objective optimization problems (e.g.^10^). In both cases, the decision maker is empowered as the uncertainty in weight definition is taken into account.

The implementation of data‐driven, or experimental, optimization differs from numerical optimization, as here the parametrization of objective functions requires results as opposed to *in silico* evaluations. This accentuates the importance of both efficacy and efficiency in data‐driven optimization. It has been demonstrated that a gridded variant of the Simplex technique meets such requirements, and more so compared to model‐driven DoE approaches, as it is successful in rapidly reaching optimal operating conditions in challenging early phase HT investigations.^11^, ^12^ In this work, this Simplex‐based approach is extended further by deploying the technique in three HT chromatography‐based case studies to optimize objective functions comprised of multiple responses. Three responses are considered simultaneously in each case study; namely, yield, residual host cell DNA content, and host cell protein (HCP) content. Each display strong nonlinear effects within the studied experimental spaces which make them challenging. To enable multi‐objective optimization, using the Simplex technique, these responses were amalgamated through the desirability approach.^13^, ^14^, ^15^ To facilitate decision making, this used the approach of including the response weights, together with the experimental inputs, in the optimization problem. In essence, the Simplex method was tasked with searching through a complex space, of both experimental conditions and response weights, for a scalar optimum in a rapid fashion. The success of the gridded Simplex technique in achieving this is compared against inferences made based on a DoE approach using regression models. Here, quartic models (i.e., of fourth order) were used to increase their compatibility with the challenging nature of the case studies and to assist the application of the DoE approach.

## Materials and Methods

### Desirability approach

The desirability approach^13^, ^14^, ^15^ merges multiple responses, for a given set of input combination, into the total desirability, *D*. It is a commonly met methodology in DoE applications offered by popular commercial software packages such as Design Expert® (Stat‐Ease, Inc., MN). In this approach, the considered, *k =* 1, 2, …, *K*, responses (*y*_*k*_) are scaled between 0 and 1 (fractions inside brackets of Eqs. 1 and 2) and are used in functions returning the individual desirabilities, *d*_*k*_. Equations 1 and 2 are applied when a response is to be maximized or minimized, respectively. In these equations, a target value *T*_*k*,_ is compared against lower (*L*_*k*_) and upper (*U*_*k*_) limits within a set of inequalities so as to enable the optimization of the *k*th response. The *w*_*k*_ exponents are weights which change the shape of *d*_*k*_ from a straight line (i.e., *w*_*k*_ = 1) to concave up or down (i.e., *w*_*k*_ lower or greater than 1, respectively). Hence, they determine the relative importance of reaching *T*_*k*_ against the alternatives. *D* is then calculated from *d*_*k*_ by Eq. 3. It is important to highlight, that Eqs. 1–3 return values in [0,1]. Furthermore, a value approaching 1 depicts a good performance as opposed to a value of 0. Hence, investigations using desirabilities typically aim to maximize *D* to identify optima.

In deploying the desirability approach, the decision maker is required to: (1) distinguish the responses between those that are to be maximized and those that are to be minimized; (2) establish the values of *T*_*k*_, *U*_*k*_, and *L*_*k*_; and (3) select *K* weights, *w*_*k*_. The first is intuitive whereas the second can fulfil the role of constraints on the individual responses. For example, in minimizing an impurity level (Eq. 2), *T*_*k*_ could coincide with the detection limit of a highly sensitive analytical method whereas *U*_*k*_ could be set based on commonly regulatory acceptable values (e.g., Ref. 16). Setting these levels creates an admissible region of conditions wherein all responses will meet end‐user requirements. The selection of weights will determine the optimal conditions based on the differences between the individual desirabilities. Thus, weight specification is a critical and challenging task for the decision maker. The complexity introduced by this requirement is not unique to the desirability approach; all response amalgamation techniques face the same challenge. However, the desirability approach delivers optima belonging to the Pareto set^17^ which is not true for other techniques based on, for example, weighted sums.^18^ This additional feature is valuable for the end‐user as obtaining a member of the Pareto front, or set, prevents the selection of a solution which is worse than an alternative in all responses.^19^, ^20^ Hence, the desirability approach yields members of the Pareto set and assists in the selection of a scalar optimum; a condition leading to an optimal composite response. This feature is considered in the deployment of the gridded Simplex method, described next.

(1) $$d_{k}=\left\{\begin{array}{ll} 1 & y_{k}>T_{k}\\ {\left(\frac{y_{k}-L_{k}}{T_{k}-L_{k}}\right)}^{w_{k}} & L_{k}\leq y_{k}\leq T_{k}\\ 0 & y_{k}<L_{k} \end{array}\right.$$

(2) $$d_{k}=\left\{\begin{array}{ll} 1 & y_{k}<T_{k}\\ {\left(\frac{y_{k}-U_{k}}{T_{k}-U_{k}}\right)}^{w_{k}} & T_{k}\leq y_{k}\leq U_{k}\\ 0 & y_{k}>U_{k} \end{array}\right.$$

(3) $$D=\sqrt[{K}]{\prod_{k=1}^{K}d_{k}}$$

### Grid compatible Simplex algorithm

The grid compatible Simplex algorithm variant^21^ allows for the experimental deployment of the Simplex optimization technique to coarsely gridded data, typical of those generated in early stage bioprocess development activities. Performing early stage studies with the gridded Simplex method entails the preprocessing of the gridded search space by assigning monotonically increasing integers to the levels of each factor and replacing any missing data points with highly unfavorable surrogate points.^11^ This is followed by the definition of a starting point, or initial simplex, and the evaluation of the experimental conditions defined by the coordinates of its vertices in the input space, *X*. The method then enters an iterative domain where it suggests test conditions for evaluation and the obtained response is then converted, by the method itself, into a new set of test conditions for evaluation. When replication is present, as it is good experimental practice, averaged responses are used. By following this process, the method optimizes a user defined objective function by moving away from unfavorable areas of a search space and focusing on the more promising experimental conditions until it identifies an optimum. A detailed account of this Simplex algorithm variant can be found in Ref. 21.

The iterative workflow encapsulates the deployment of the gridded Simplex method where actions and the completion of experiments take place in real time. In this work, however, the Simplex method was deployed on an already evaluated grid of test conditions (i.e., all conditions were evaluated in brute force before Simplex deployment). While this deviates from its intended use, it serves to simulate the deployment of the technique, had the data not already been generated. At the same time, it allows for a thorough assessment of performance and an unbiased comparison against more common regression analysis‐based DoE approaches.

### Case studies

Three case studies are presented here with data originating from full factorial HT investigations of chromatographic separations. These are binding studies using the filter plate format, which is typically selected for such applications (the method can, however, be applied to investigations involving alternative HT formats such as miniature columns^12^). Ion exchange and multimodal resins were considered in these studies, run in flowthrough mode. They aim to evaluate the impact of three inputs on three outputs comprised of the yield, and the residual host cell DNA and HCP contents. A summary of the case studies can be found in Table 1. Their experimental details are given in the following section.

**Table 1:** Details of Case Studies 1–3

Case Study	1	2	3
Product	mAb (IgG1, pI 8.4)	mAb (IgG4, pI 6.7)	mAb (IgG1, pI 8.4)
Chromatography mode	Anion exchange	Anion exchange	Multimodal
Grid conditions	48 (duplicated)		
Inputs	Levels		
A: pH	6.9, 7.2, 7.5, 7.8	6.0, 6.5, 7.0, 7.5	5.8, 6.8, 7.3, 7.8
B: Load (g L^−1^)	100, 150, 300	50, 125, 175	75, 150, 300
C: Conductivity (mS cm^−1^)	2.5, 4.0, 6.0, 10.0	3.4, 5.0, 7.0, 10.0	3.5, 7.0, 14.0, 25.0
Responses			
(1): *y*_*1*_	%Yield		Maximize
(2): *y*_*2*_	Host Cell Protein content (ppm) (HCP)		Minimize
(3): *y*_*3*_	Host Cell DNA content (ppb) (DNA)		Minimize
Weights of responses			
(1): *w*_yield_	0.5, 1.0, 2.0		
(2): *w*_HCP_	0.5, 1.0, 2.0		
(3): *w*_DNA_	0.5, 1.0, 2.0		

#### Experimental Details for Case Studies 1–3

High throughput experiments were conducted on a Tecan Evo® 200 liquid handling system (Tecan Systems, Inc., San Jose, CA). 96‐well AcroPrep™ filter plates (P/N 5065, Port Washington, NY) were used for these studies with a pore size of 1.2 μm. These were filled with process intermediates diluted to a concentration of 1.14 g L^−1^ in 700 μL volumes and at buffering conditions specified in Table 1. The load conditions in Case Studies 1 and 3 were comprised of an IgG1 isotype mAb (pI 8.4). Case Study 2 used a load condition with an IgG4 mAb (pI 6.7). The desired conductivity was achieved with NaCl additions. Resin volumes in each well were varied from 2.7 to 16 μL to obtain the desired resin loadings between 50 and 300 g L^−1^ (Table 1). Both Case Studies 1 and 2 used the Poros™ 50 HQ anion exchange resin (Thermo Fisher Scientific, Waltham, MA). Case Study 3 used the multimodal resin, Capto™ Adhere (GE Healthcare, Piscataway, NJ). Upon resin addition and loading, the plates were agitated for 60 min at 1,250 rpm. This was followed by flowthrough fraction collection in 96‐well plates via vacuum filtration. The analysis of these fractions involved yield determination with a reverse phase HPLC assay.^22^ Briefly, a gradient from 20 to 57% Buffer B was run for 55 column volumes. Buffer A was 0.1% TFA in water and Buffer B was 0.1% TFA in acetonitrile. HCP was quantified using a Cygnus ELISA assay kit (P/N F640, Cygnus Technologies, Southport, NC) with a minimum detection at 30 ppm. DNA content was determined using a PicoGreen® dye (Thermo Fisher Scientific) and fluorescence measurements with excitation and emission wavelengths at 480 and 520 nm, respectively.

### Data analysis

#### Regression Analysis

The regression analysis DoE‐based approach was applied here, along with the desirability technique, in a usual fashion. Experimental data were used to fit regression models which were then used to obtain predicted response values for the different experimental conditions. Once available, the predicted responses (i.e., ${\hat{y}}_{k}$) were amalgamated through the desirability approach (Eqs. 1–3) to yield predicted total desirabilities. These were used to capture trends and identify operating conditions leading to the most favorable overall performance based, essentially, on model predictions. Experimental studies through DoE methodologies use, typically, designs allowing for the unbiased estimation of up to second‐order model terms. Here, however, the trends in the experimental data were highly nonlinear and hence high‐order model terms were required to improve the likelihood of a successful application of the DoE approach. Consequently, models including up to 4th order terms (quartic) were considered, similar to those tested in Ref. 23. The structure of these models supported the consideration of complex nonlinear effects. Their analysis was carried out in MATLAB 2014a (The MathWorks® Incorporated, MA) with stepwise regression^24^ using *P*‐values of 0.05 and 0.1 for adding and removing, respectively, terms from an intermediate model. Different initial model structures were postulated prior to reduction and final model acceptance took place by selecting the one leading to the lowest root mean square error. The derived models were used to enable accurate point estimations and hence no model hierarchy was maintained. The fitting of such models was made possible using all available experimental data in the model building process (excluding missing data). While this resembles a brute force approach, it is important to underline that all inferences were made based on model predictions as it would occur in an investigation using typical designs such as composite designs.

To obtain a rigorous understanding about the performance of the described DoE approach, the aforementioned procedure was applied multiple times to generate a population of results. To achieve this, three weight values were examined per response (Table 1) the combination of which returned *i =* 1, 2, …, 27 unique sets, or triplets, of weights. Hence, for the *i*th set of weights, a predicted total desirability was obtained (i.e., ${\hat{D}}_{i}$) the maximum of which corresponded to a predicted optimum, ${\hat{x}}_{i*}$, in the gridded experimental space, *X*, for the given case study. The performance of the DoE approach was then quantified for each case study by comparing the model derived ${\hat{D}}_{i}$ and ${\hat{x}}_{i*}$ to their respective counterparts, *D*_*i*_ and *x*_i*_ which were both obtained based on the raw experimental data. The comparison used two metrics: (1) the Pearson correlation coefficient^25^ between *D*_*i*_ and ${\hat{D}}_{i}$, $r_{D_{i},{\hat{D}}_{i}}$ and; (2) the Euclidean distance between *x*_i*_ and ${\hat{x}}_{i*}$, $\lambda_{x_{i},{\hat{x}}_{i*}}$. The former describes the ability of the models to make predictions capturing the trends in *D*_*i*_, as it is only affected by random error^25^ and is not to be confused with the nonparametric Spearman's rank correlation coefficient,^26^ *ρ*, used here to assess trade‐offs between responses. Typically, a $r_{D_{i},{\hat{D}}_{i}}\geq \sqrt{0.60}$ depicted an acceptable performance in capturing trends in the data whereas a $r_{D_{i},{\hat{D}}_{i}}\geq \sqrt{0.95}$ indicated that almost the entirety of the data trends were captured.^27^ Conversely, the distance $\lambda_{x_{i},{\hat{x}}_{i*}}$, which was also calculated by assigning monotonically increasing integers to the levels of each factor in *X* (*Grid compatible Simplex algorithm* section), was used to account for the iterative workflow in DoE studies wherein a design space is moved based on the results of earlier studies. Within the context of DoE, and the analysis presented here, if a model missed the optimum *x*_i*_, the likelihood of including it within the search space of a follow‐up study increases as $\lambda_{x_{i},{\hat{x}}_{i*}}$ decreases from $\sqrt{2.0}$ (the length of a face diagonal in a unit cube). Hence, a $\lambda_{x_{i},{\hat{x}}_{i*}}\leq \sqrt{2.0}$ meant that a follow‐up study would be highly likely to reach the optimum, even if an earlier study had missed it. The 27 values of $r_{D_{i},{\hat{D}}_{i}}$ and $\lambda_{x_{i},{\hat{x}}_{i*}}$, one each for a set of weights, were then used in inequalities, described in detail in the Supporting Information, to obtain a success rate of the DoE approach per case study. This was based on the ratio of instances wherein the inequalities were met over the total number of instances (i.e., 27). These results represented the general suitability of a regression analysis DoE‐based approach per case study for a large number of specified weights and in the presence of stringent and lenient performance requirements.

#### Simplex Method

The deployment of the Simplex method in the three case studies took place as described in the *Grid compatible Simplex algorithm* section and it used the averaged measured total desirabilities, ${\bar{D}}_{i}$ for each of the aforementioned 27 sets of weights on the three responses (*Regression analysis* section). A traditional application of the Simplex method would involve the deployment of the technique to identify optima in each case study and for each set of weights. Here, however, to make the weights part of the optimization problem itself, the three‐dimensional gridded input space, *X*, was expanded to the gridded space *XW* according to Scheme 1 (Supporting information also including an executed example) in an additional set‐up step prior to method deployment for each case study. Likewise, all ${\bar{D}}_{i}$ were joined in a single overall desirability, *D*_*T*,_ which was the objective function to be maximized. This is similar to the implemented manipulations in Ref. 12 and as a result the Simplex method was deployed in each case study to identify, simultaneously, the operating condition and set of weights leading to the maximum desirability value in *D*_*T*_ denoted as xw_*_. This is the global scalar optimum which was Pareto optimal and at the same time it delivered the best and most balanced performance, as depicted by the user‐defined criteria (i.e., weights), within the admissible, due to the constraints imposed by *U*_*k*_ and *L*_*k*_ (*Desirability approach* section), region of the three responses in a case study. This approach is similar to the deployment of the Simplex method in Ref. 28 who used the interactive desirability function approach of^29^ and is made possible since Eqs. 1–3 return values that all belong to the same scale. Here, however, the weights, or preference parameters, became inputs of the optimization problem and were adjusted in an unsupervised fashion by the Simplex method itself instead of implementing this manually.

Similar to the DoE‐based approach, success rates were also calculated by obtaining a population of results through the deployment of the Simplex method from 300 different and randomly selected starting points. For each of these searches, the reached optimum was compared against xw_*_ and when they coincided a given search was deemed to converge. Conversely, if the reached optimum did not coincide with xw_*_, but the corresponding grid location belonged to the Pareto set (i.e., no other condition within the search space was better in all three responses) and led to a *D*_*T*_ value within 1% to the one at xw_*_, then the loss in the overall performance was not critical. Hence, in this case the method was deemed to reach to a near optimal condition with a highly attractive performance across all three responses. In all other cases, the method did not deliver an acceptable operating condition. For the assessment of the Simplex method's efficiency, in this multi‐objective optimization application, each of the aforementioned searches was tracked so as to determine the relationship between the number of the evaluated, unique, test conditions in *X,* and the improvement in the objective function, *D*_*T*_. It needs to be clarified that the used weights of the three responses (Table 1), in all three case studies, were found to lead to situations where the shapes of the individual desirabilities, as a function of the responses, matched the expected behavior (i.e., linear, concave up and down) ensuring that the chosen range was of interest. A wider range was also considered and while it led to more pronounced shapes in the individual desirabilities, it did not affect the location of xw_*_.

The Simplex method was encoded for objective function maximization in MATLAB 2014a (The MathWorks® Incorporated) on a dual Intel Xeon E5‐2650 CPU workstation with 32 GB of RAM running Windows Server 2003 (Microsoft Corporation, WA). The application of the method with the Parallel Computing Toolbox (The MathWorks) allowed for the parallel deployment of 30 simplex searches, with each lasting, typically, well below a minute in real time. Regression analysis was implemented using standard functions supplied with MATLAB's Statistics and Machine learning Toolbox.

## Results and Discussion

### Data Trends and Pareto Optimality

**Figure 1:**
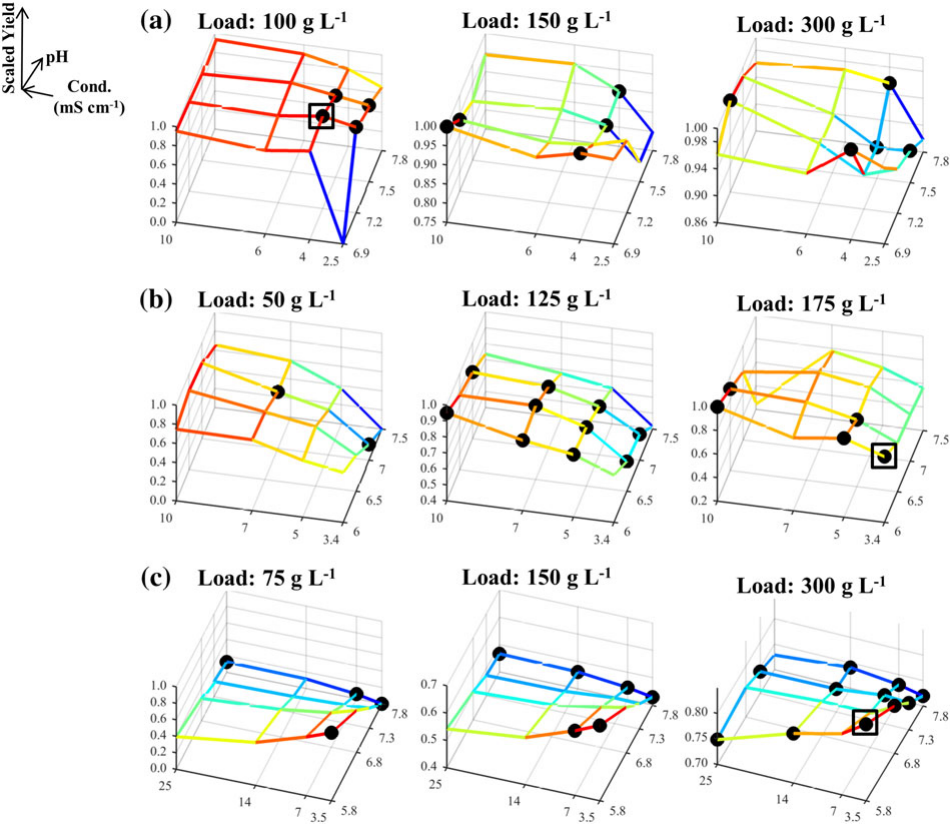
Mesh plot of scaled yield, based on the fraction inside the brackets of Eq. 1, for Case Studies 1–3 in (A)–(C), respectively. (●) Members of the Pareto front; (□) global scalar optimum in the conductivity, pH, and load input space. Blue color corresponds to low yields whereas red color corresponds to high yields.

**Figure 2:**
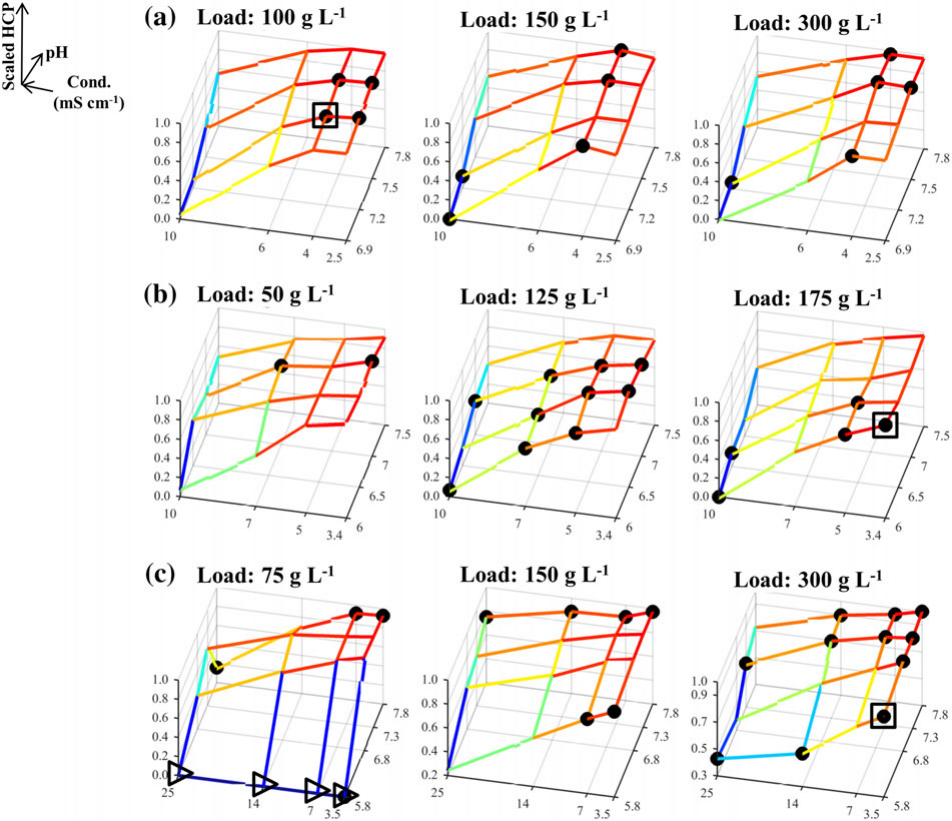
Mesh plot of scaled HCP content, based on the fraction inside the brackets of Eq. 2, for Case Studies 1–3 in (A)–(C), respectively. (●) Members of the Pareto front; (▹) missing data points (replaced by a surrogate); (□) global scalar optimum in the conductivity, pH, and load input space. Blue color corresponds to high HCP content whereas red color corresponds to low HCP content.

**Figure 3:**
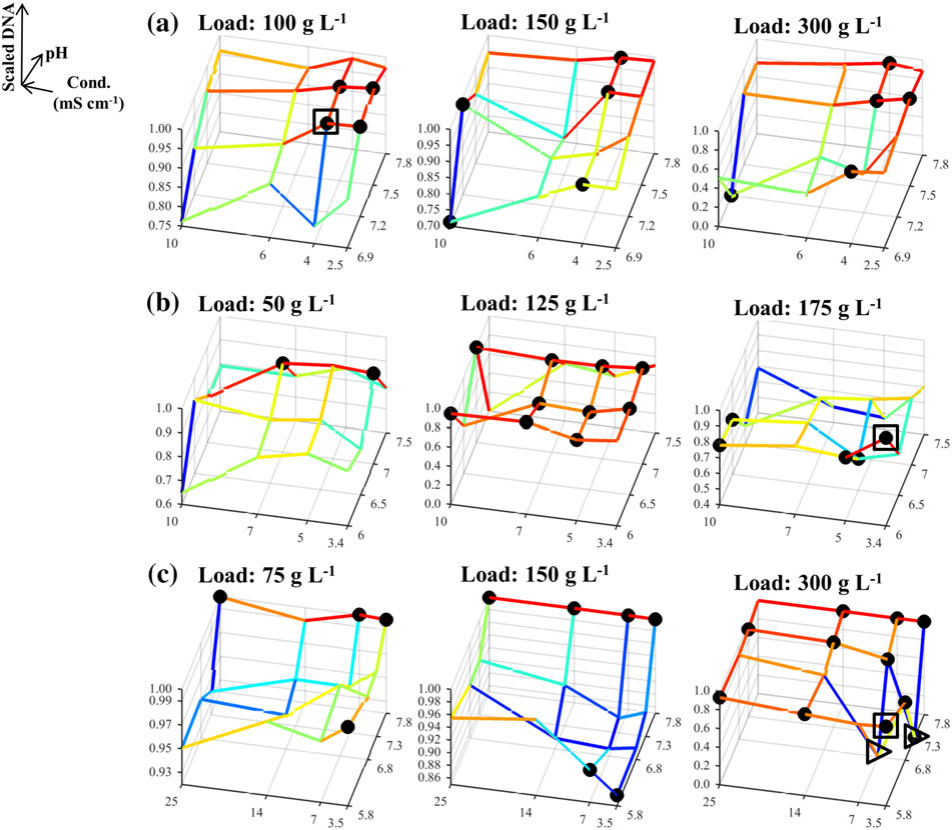
Mesh plot of scaled host cell DNA content based on the fraction inside the brackets of Eq. 2, for Case Studies 1–3 in (A)–(C), respectively. (●) Members of the Pareto front; (▹) missing data points (replaced by a surrogate); (□) global scalar optimum in the conductivity, pH, and load input space. Blue color corresponds to high DNA content whereas red color corresponds to low DNA content.

The trends in the experimental data from the three case studies can be observed in Figures 1, 2, 3 for the average yield, HCP and DNA contents, respectively. In these figures, the responses have been scaled to take values between 0 and 1, as described in the *Desirability approach* section (value of 0 is least favorable whereas value of 1 is most favorable). The results from Case Studies 1 and 2 were in general agreement with the behavior expected from running anion exchange resins in flowthrough mode; buffer conditions are preferred which increase the yield by diminishing electrostatic interactions (i.e., high *Conductivity* and a *pH* lower than the product's pI) which could lead to a significant binding of the product to the resin (Figures 1A,B). Conversely, a higher purity in the flowthrough pool was reached through the enhancement of the aforementioned interactions between the anionic impurities and the resin (e.g., low *Conductivity*). Here, the latter trend is more evident for the clearance of HCP (Figures 2A,B) than it is for the removal of residual host cell DNA (Figures 3A,B) for both Case Studies 1 and 2. The trends in the DNA clearance data for Case Studies 1 and 2 appear to be highly nonlinear with respect to all three inputs (i.e., *pH*, *Load*, and *Conductivity*). The complexity of these dependencies is greater for Case Study 2 than for Case Study 1 because here the tested *pH* range also included the pI of the protein which shifted the competition for the resin's binding sites in favor of the product at high *pH* values. This led to a decreased DNA clearance (Figure 3B) with small effects on the HCP clearance (Figure 2B). Furthermore, the dependency between the *Load* and the HCP clearance was weak for both Case Studies 1 and 2 (Figures 2A,B), whereas increasing the *Load*, in these flowthrough anion exchange steps, returned a further increase in the yield (Figures 1A,B). This can be attributed to the displacement of the weakly bound product by competing solutes. The multimodal chromatography step in Case Study 3, also ran in flowthrough mode, showed yield trends (Figure 1C) that were significantly different to those in Case Studies 1 and 2; increased yield is achieved by decreasing the *Conductivity* whereas the *Load* has a considerably stronger positive effect. The dependency between the inputs and the HCP content (Figure 2C) was similar to the one observed in Case Studies 1 and 2. Similar trends were also observed for the removal of the DNA impurity, in terms of the observed complexity, which appeared to be influenced by high‐order interactions including the *Load* input (Figure 3C).

**Figure 4:**
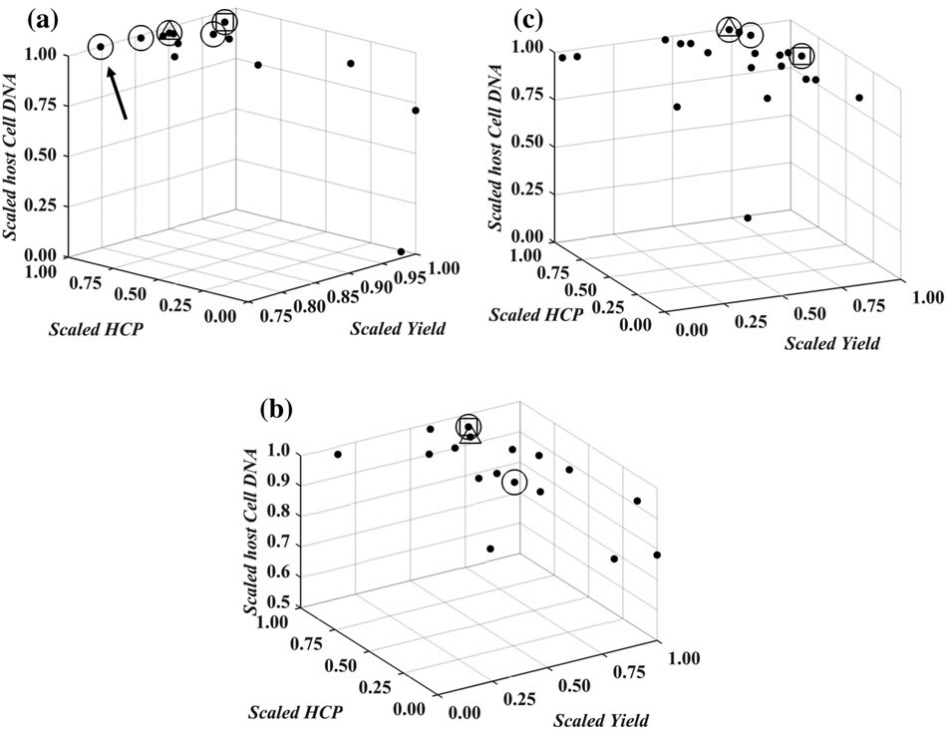
Pareto fronts for Case Studies 1–3 in (A)–(C), respectively. (A)–(C) Show scaled (based on the fractions inside the brackets of Eqs. 1 and 2) responses for yield, host cell DNA and HCP content. (●) Members of the front; (○) grid conditions belonging to the front; (□) global scalar optimum in the conductivity, pH, and Load input space; (▽) Simplex‐derived local optimum in the conductivity, pH and load input space. In (A)–(C), a value of zero is the least favorable (i.e., low yield, high HCP, and DNA content) whereas a value of 1 is the most favorable (i.e., high yield, low HCP, and DNA content). Arrow denotes a condition with a small improvement in DNA clearance accompanied by a significant decrease in yield.

The data trends in Figures 1, 2, 3 return a visual confirmation of the challenging task of identifying windows of operation wherein high yields are returned along with low impurity contents. This accrues from the existence of the nonlinear trends, for each of the individual responses, and trade‐offs. These are made apparent, for example, by comparing Figures 1B and 2B. Here, high yields are accompanied by high HCP content in the flowthrough pool. More information about these trade‐offs can be obtained by the Pareto fronts in Figure 4. The Pareto front for Case Study 1 (Figure 4A) differed to those of the remaining case studies since its members lied predominantly on the upper edges of the formed cube. Hence, a good clearance of host cell DNA was accompanied by a good clearance of HCP and medium to high yields whereas exceedingly high yields resulted in a significant deterioration in the achieved clearance of both DNA and HCP. This behavior accrues from the existence of both positive (i.e., HCP/DNA content with *ρ* = 0.5776 and *P*‐value <0.0001) and negative (i.e., yield/DNA, with *ρ* = −0.6000 and *P*‐value <0.0001, and yield/HCP content with *ρ* ≈ −0.5646 and *P*‐value <0.0001) correlations between the responses. In Case Study 2, the front (Figure 4B) provided evidence for the negative relationship between yield and HCP, as also shown in Figures 1A and 2A (*ρ* = −0.6464 and *P*‐value <0.0001), and the weak dependence for the yield/DNA and HCP/DNA pairs (i.e., insignificant correlations for both pairs) as in both cases widely differing values of DNA content were obtained for a wide range of achieved yields and HCP clearance. A similar behavior was also displayed in Case Study 3 (Figure 4C) where the role of HCP content is analogous to the role of DNA content; weak dependencies were observed between HCP content, and both yield and DNA content (i.e., insignificant correlations in both cases) whereas increases in yield led to a less successful clearance of DNA (*ρ* = −0.6720 and *P*‐value <0.0001).

#### Data Complexity

**Figure 5:**
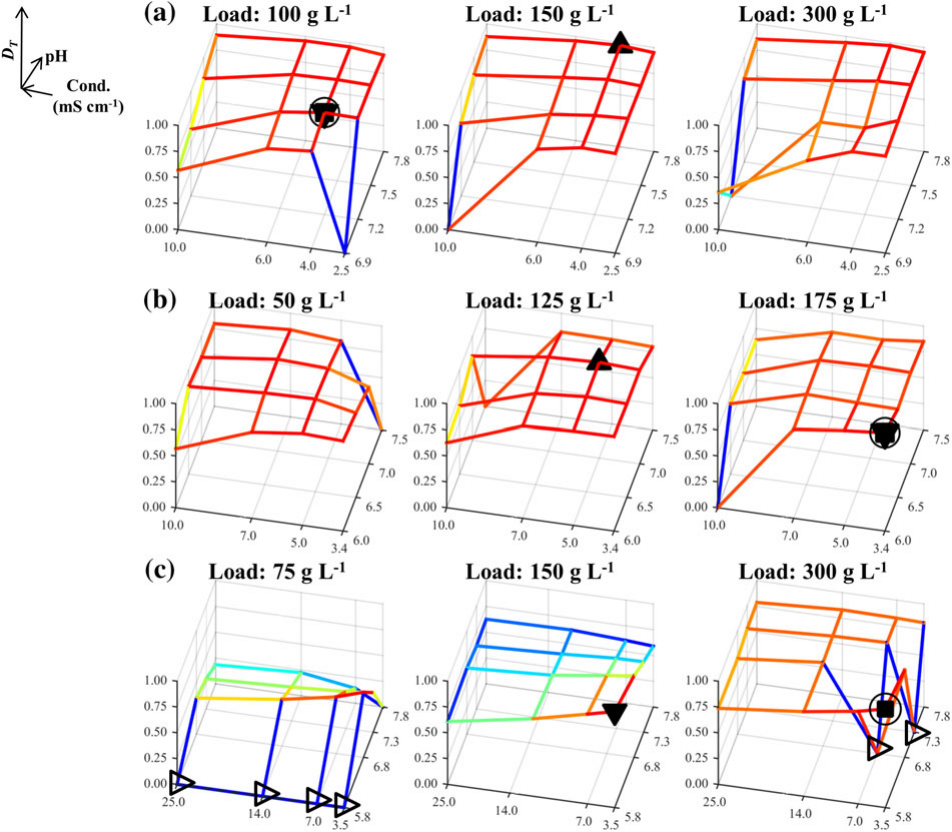
Mesh plot of concatenated averaged measured total desirabilities, *D*_*T*_, for Case Studies 1–3 in (A)–(C), respectively for a selection of weights on the three responses (detailed results can be found in Figures S1–S3 in the supporting information). In (A) and (B), the weights for the yield, HCP and DNA content responses were set to 0.5, 0.5, and 0.5 respectively whereas in (C) the weights were set to 2.0, 0.5, and 0.5 respectively. In each of (A)–(C): (○) denotes total desirability optima per set of weights based on the averaged raw measurements; (▪) denotes the global scalar optimum (also Simplex‐derived best condition); (▴) denotes the Simplex‐derived local optimum; (▾) denotes the predicted optima per set of weights returned by the regression‐based analysis approach In (C), (▹) denote missing data points (replaced by a surrogate). Blue color denotes low total desirability and red color denotes a high total desirability.

The densely populated Pareto fronts in Case Studies 1–3 can improve the chances of an investigation to identify a Pareto optimal condition in a gridded space. At the same time, however, this can render the identification of the global scalar optimum, xw_*_, (*Simplex method* section) a challenging task. In Figures 1, 2, 3, 4, □ shows the grid location leading to the maximal *D*_*T*_ value for a given set of weights on the responses in the input space *X* whereas in Supporting Information Figures S1–S3 □ shows the exact location of xw_*_ in the six dimensional *XW* space. Since Supporting Information Figures S1–S3 contain a large number of surfaces, a sub‐set of important results is shown in Figure 5. The Pareto fronts in Figure 4 indicate that the xw_*_ optima for each case study are highly favorable conditions wherein good performance is obtained across all three responses. This highlights the importance of the consideration of multiple weights for countering situations wherein processes with skewed performance are obtained due to a specification of weights which is in discord with the behavior of the studied system. For example, in Case Study 1 xw_*_ had a scaled yield of ~0.96 and both scaled HCP and DNA contents of >0.95 since further increases in any of the responses would result in an overall deteriorated performance. This is in contrast to the Pareto optimal condition with the best clearance of HCP (shown by arrow in Figure 4A) as a ~0.05 scaled increase was accompanied with a decrease of ~0.2 units in scaled yield.

The identification of xw_*_ can be complicated further by the existence of nonlinear effects which could separate it from the remaining members of the Pareto set. For example, in Case Study 2 most of the Pareto optimal conditions corresponded to a *Load* of 125 g L^−1^ but for this case study xw_*_ had a *Load* set at 175 g L^−1^ (□ in Figures 1B, 2B, and 3B). Similarly, in Case Study 1, xw_*_ had a *Load* of 100 g L^−1^ (□ in Figures 1A, 2A, and 3A) as opposed to the majority of the Pareto optimal conditions being concentrated on the upper two levels of the *Load* input. These observations serve to underline further the complexity of the data and the challenging nature of the multi‐objective optimization problems posed by the three case studies. They are, therefore, ideally suited to assess the performance of the Simplex method in not only identifying a member of the Pareto front, but instead its capacity to locate Pareto optimal conditions which also have a balanced performance across all outputs. The success of the method, along with the returned efficiency, is described next.

### Deployment of grid compatible Simplex method

#### Identification of Optima

The responses from each case study were combined based on the desirability approach for a total of 27 sets of weights (Table 1) as described in the *Simplex method* section. The obtained average total desirabilities, for each of the tested sets of weights, ${\bar{D}}_{i}$, are depicted via mesh plots in Supporting Information Figures S1–S3 for Case Studies 1–3 respectively whereas, as mentioned previously, Figure 5 shows a sub‐set of important results. The considered weights of the responses returned 5, 2, and 3 unique grid locations (○ in Figure 4 and Supporting Information Figures S1–S3) leading to the highest ${\bar{D}}_{i}$ for a given set of weights per case study respectively (*x*_i*_). In the first case study, the five grid points (Supporting Information Figure S1) spanned a wide range in all three inputs whereas in Case Study 3 the three points (Supporting Information Figure S3) spanned wide ranges across the *pH* and *Conductivity* inputs but were concentrated at a *Load* level of 300 g L^−1^. This is in agreement with the trends displayed in Figures 1C, 2C, and 3C (i.e., the majority of the Pareto optimal conditions have a *Load* set to 300 g L^−1^). Finally, in Case Study 2 the two grid locations (Supporting Information Figure S2) were adjacent to each other and not part of the majority of the Pareto optimal points as the latter had a *Load* of 125 g L^−1^ (Figures 1B, 2B, and 3B). This trend was attributed to the fact that they offered good performance across all three responses (Figure 4B).

The data depicted in Supporting Information Figures S1–S3 also show the spaces searched by the Simplex method (i.e., *XW* space with the *D*_*T*_ response) for the identification of the optimal coordinates in the *pH*, *Load*, and *Conductivity* input space, and the favorable weighting of the three responses. As mentioned in the *Simplex method* section, the method was initialized from 300 different and random starting points to assess in detail its likelihood to converge to optima and to ensure its unbiased deployment. In the second case study, 92.3% of these searches reached to xw_*_ (▪ in Figure 5B). This was reached to by 63.7 and 48.3% of these searches for Case Studies 1 and 3, respectively (▪ in Figures 5A,C respectively). The remaining of the searches converged to a single local optimum for Case Studies 1 and 3 (▽ in Figures 5A,C respectively). For Case Study 2, while multiple local optima (▽ in Supporting Information Figure S2) were also reached, only one of them was predominant (i.e., ~6.5% of the searches vs. a single search for the rest as indicated by the arrows in Supporting Information Figure S2, with the dominant local optimum also depicted by ▽ in Figure 5B). For all case studies, the identified local optima belonged to the corresponding Pareto set (▽ in Figure 4 showing the grid location of a local optimum for a given set of weights on the responses in the input space *X*) and, with the exception of Case Study 2, they also coincided with *x*_i*_.

**Figure 6:**
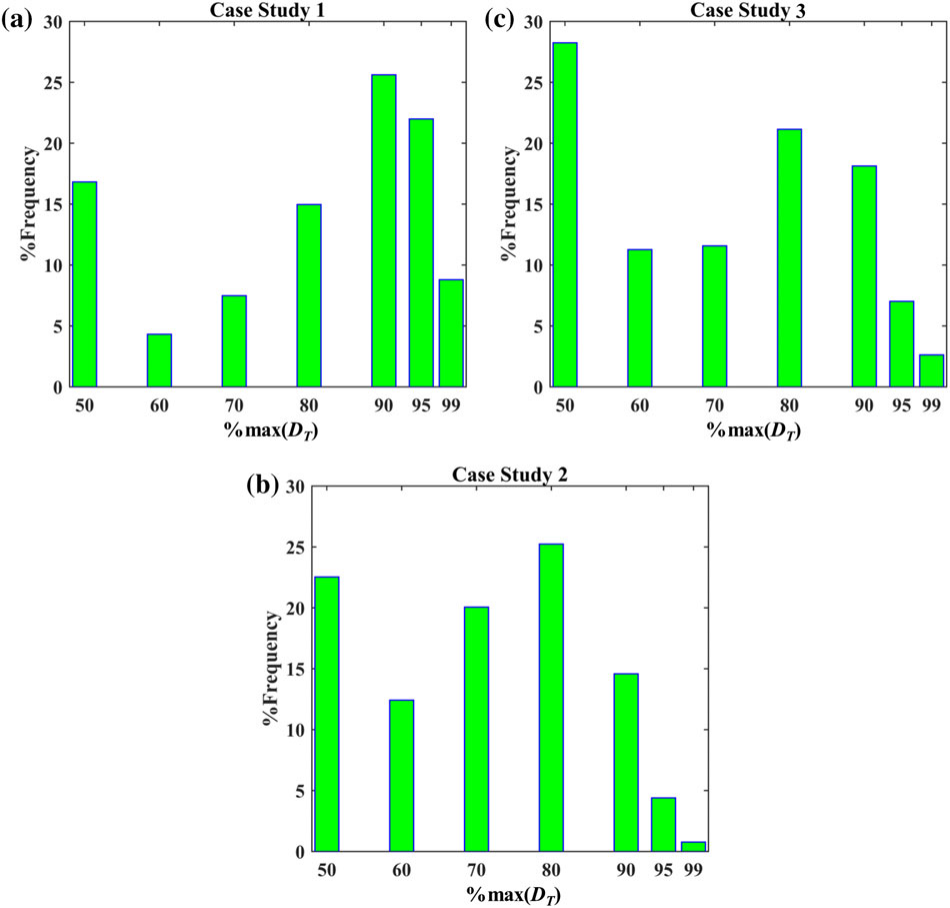
Distribution of normalized total desirability across all considered weights (i.e., %max(***D***_***T***_)) for Case Studies 1–3 shown in (A)–(C), respectively.

The lowest achieved convergence to xw_*_ for Case Study 3 can be attributed, primarily, to the inclusion of missing data (▹ in Figures 1C, 2C, 3C, and 5C and Supporting Information Figure S3) which make the planar polygon formed in the *pH*/*Conductivity* plane at the upper *Load* level concave in close proximity to xw_*_ (▪ in Figure 5C). This resulted in its shielding from the Simplex method forcing it to follow only limited trajectories in the searched space because the optimum lied on a boundary of the *pH*, *Conductivity*, and *Load* space. Despite this, the local optimum (▽ in Figure 4C) led to a performance which was within 1% of the maximal *D*_*T*_ value and hence it posed a viable alternative operating condition. The same applied for the local optimum reached to by the Simplex method for Case Study 1. Here, the local optimum (▽ in Figure 4A) was characterized by a decrease of ~0.07 units in scaled yield and an increase of ~0.05 and ~0.02 units in scaled HCP and DNA content, respectively. Consequently, for both Case Studies 1 and 3, the Simplex method delivered either the global scalar optimum or a near optimal condition. This is supported further for Case Studies 1 and 3 by Figures 6A,C. The Simplex method reached to a condition with such a similar performance to xw_*_ even when only ~9% and ~3% of the *XW* conditions in Supporting Information Figures S1 and S3, respectively, had *D*_*T*_ values within 1% of the one at xw_*_. The method, however, failed to deliver an acceptable operating condition when it converged to the predominant local optimum of Case Study 2. Figure 4B shows that this condition (▽) led to a low improvement in terms of DNA clearance (i.e., <0.01 unit) and significant loss in yield (~0.05 scaled units) and HCP clearance (~0.1 scaled unit) compared to the global scalar optimum (□). However, taking into account the dense Pareto set (Figure 4B) and the fact that only ~1% (Figure 6B) of the *XW* conditions (Supporting Information Figure S2) led to a *D*_*T*_ value which was within 1% of the one at xw_*_, leads to the conclusion that the Simplex method can be considered to be highly successful for this case study as well. The results in Figure 6 also provide an additional description of the complexity of the response surfaces in Supporting Information Figures S1–S3.

#### Simplex Method Efficiency

**Figure 7:**
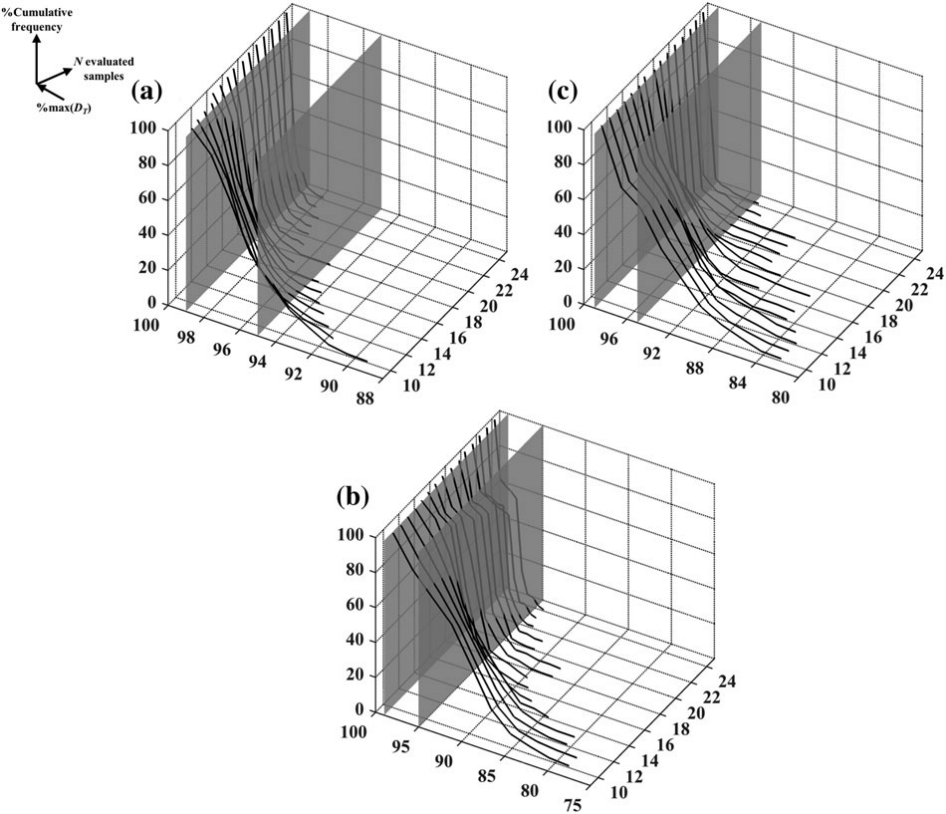
Improvement of objective function for 10–24 simplex‐based experiments across all 300 searches for Case Studies 1–3 in (A)–(C), respectively. The shaded patches correspond to 95 and 99% of the maximum of total desirability across all considered weights (i.e., %max[***D***_***T***_]) for each case study.

While the deployment of the Simplex method to Case Studies 1–3 was found to be successful in identifying optima, it was also important to assess the efficiency with which these were reached. This information is presented in Figure 7. Here, the cumulative frequency of the number of searches achieving a given performance, expressed as a percentage of the *D*_*T*_ value obtained at xw_*_ per case study, is examined as a function of the number of the evaluated grid conditions in *X*. The displayed behavior demonstrates that, as expected, allowing the Simplex method to continue searching the investigated experimental space led to a convergence at grid locations with improved overall performance. The selection of up to 24 conditions was considered (i.e., 50% of the conditions in the *pH*, *Conductivity*, and *Load* input space, *X*) and this was found to be sufficient for at least ~95% of the simplex searches to reach conditions with *D*_*T*_ values within 5% of the one at xw_*_ for all case studies. This also serves to underline the fact that, due to its nature, the method will increasingly focus its evaluations in the more favorable areas of the underlying response surface culminating to the encirclement of an optimum. These deliver a description of its robustness, as shown in Ref. 11 in the coarse grids used during early stage process development investigations. Conversely, reaching grid locations with *D*_*T*_ values which were within 1% of the one at xw_*_ is more challenging. For Case Study 1 (Figure 7A) such searches comprised ~90% of the population of the 300 searches, whereas for Case Studies 2 and 3 (Figures 7B,C) these comprised ~35 and ~40% of the searches, respectively. Likewise, the diametrically opposite situation of considering only 10 conditions, led to a very low number of searches reaching to such favorable locations. Here, it needs to be clarified that in the deployed *XW* space, an initial simplex had seven vertices and thusly a search with 10 evaluated conditions is one that has selected only three additional conditions to those of the starting point. Taking this into account, and the fact that ~70, ~25, and ~45% of the searches reached to conditions with *D*_*T*_ values within 5% of the maximum, for Case Studies 1–3 respectively, demonstrate that the Simplex method can lead to a condition with an improved performance very rapidly.

### Deployment of high‐order regression models

**Figure 8:**
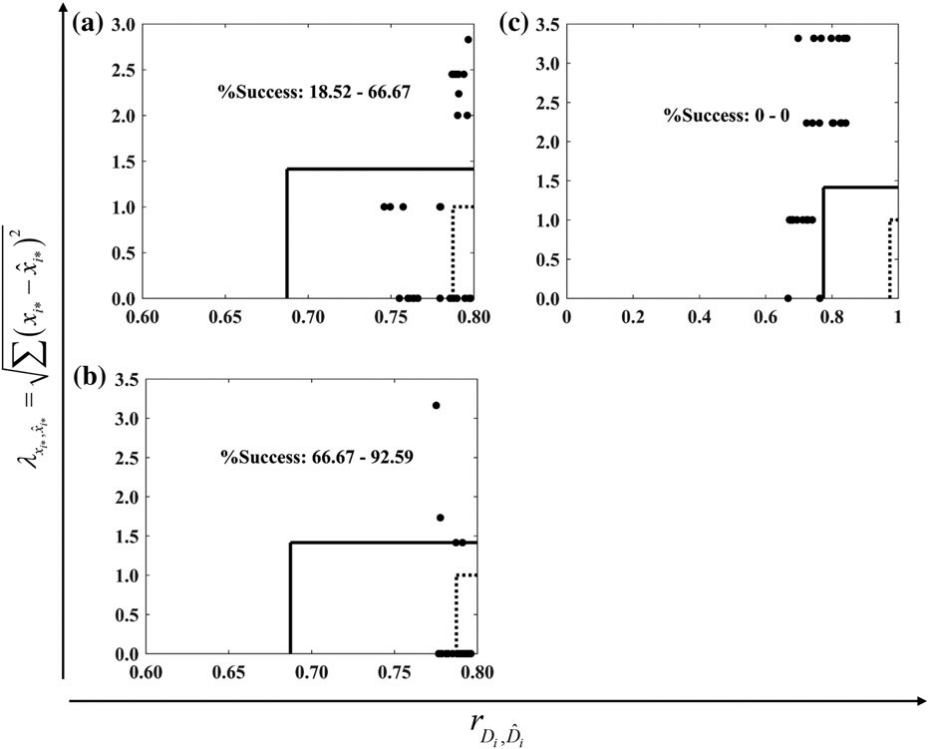
Success of a regression‐based approach for Case Studies 1–3 in (A)–(C), respectively. The *x*‐axis shows the Pearson correlation coefficient between the measured and predicted desirabilities for each set of the *i* = 1, 2, …, 27 weights, $r_{D_{i},{\hat{D}}_{i}}$. Likewise, the *y*‐axis shows the Euclidean distance between the measured and predicted optima per set of weights, $\lambda_{x_{i},{\hat{x}}_{i*}}$. (−) defines an acceptance region with low requirements (i.e., a $\lambda_{x_{i},{\hat{x}}_{i*}}$ of at most $\sqrt{2.0}$ and $r_{D_{i},{\hat{D}}_{i}}$ of at least $\sqrt{0.6}$. (−) defines an acceptance region with high requirements (i.e., a of at most 1.0 and a $r_{D_{i},{\hat{D}}_{i}}$ of at least $\sqrt{0.95}$).

To assess further the performance of the Simplex method, a comparison is made here with a DoE approach using fourth order regression models. These were calibrated and applied as described in the *Regression analysis* section. The obtained models are shown in the Supporting Information, along with the predicted individual desirabilities (Supporting Information Figures S4–S6). As mentioned in the *Regression analysis* section, the success of this approach per case study was assessed by calculating $r_{D_{i},{\hat{D}}_{i}}$ and $\lambda_{x_{i},{\hat{x}}_{i*}}$ for each set of the 27 weights and by comparing them against threshold values. The results of these comparisons are shown graphically in Figure 8.

The deployment of the quartic models was severely challenged by the data when the most stringent requirements were posed on $r_{D_{i},{\hat{D}}_{i}}$ and $\lambda_{x_{i},{\hat{x}}_{i*}}$ (*Regression analysis* section). In this case, the models were unsuccessful for both Case Studies 1 and 3 (18.52 and 0% in Figures 8A,C respectively) and with a limited success for Case Study 2 (66.67% in Figure 8B). Imposing less stringent success criteria (*Regression analysis* section) for both $\lambda_{x_{i},{\hat{x}}_{i*}}$ and $r_{D_{i},{\hat{D}}_{i}}$ in Case Study 3 did not lead to an improvement in the performance of the regression model‐based approach (Figure 8C). Here, while cases existed wherein *x*_i*_ and ${\hat{x}}_{i*}$ coincided, the predictions of the model cannot be considered to be reliable since $r_{D_{i},{\hat{D}}_{i}}$ values were obtained which could be as low as ~0.65. The opposite trend was also observed in Figure 8C as improved correlations (i.e., $r_{D_{i},{\hat{D}}_{i}}>0.80$) led to less well approximated optima based on the model predictions (i.e., $\lambda_{x_{i},{\hat{x}}_{i*}}>2.0$). This is made more apparent for Case Study 1 (Figure 8A) where model predictions, which correlated almost perfectly with the made measurements (i.e., $r_{D_{i},{\hat{D}}_{i}}>0.95$), resulted in wrongly identified optima (i.e.,$\;\lambda_{x_{i},{\hat{x}}_{i*}}>2.0$), limiting therefore the success of the approach for this case study. This behavior is similar to the one observed in Ref. 11 Conversely, in Case Study 2 (Figure 8B), the relationship between $\lambda_{x_{i},{\hat{x}}_{i*}}$ and $r_{D_{i},{\hat{D}}_{i}}$ is synergistic since here strong correlations also led to accurate determination of the optimum. Hence, the best performance of the regression model‐based approach was obtained for Case Study 2. Together, these observations led to the conclusion that the regression approach had low success across the presented case studies.

### Evaluation of the Simplex method‐based approach for multi‐objective optimization

#### Concurrent Determination of Weights and Operating Conditions Via the Grid Compatible Simplex Method

Including the weights of the desirability approach in the optimization problem was found to be of value in these multi‐objective optimization case studies. As shown in Ref. 17 and supported by Figure 4, the desirability approach by itself returns optima that are members of the Pareto set. While this offers protection against choosing conditions being entirely suboptimal, the decision maker is still required to make a choice regarding the most attractive condition because not all Pareto set members will be interchangeable (*Identification of optima* section for Case Studies 1 and 2). Hence, since the desirability approach yields Pareto set members, using a deterministic selection of weights could lead to operating conditions not reflecting the true performance of the investigated unit operation. An approach based on mathematical models is less affected by this since, assuming that a model is fit for purpose, its predictions can be used to investigate any weight combination (e.g., Ref. 30). The development of models, which can be used for such a task is not trivial (*Deployment of high‐order regression models* section), nor is it guaranteed (e.g., Supporting Information for Case Study 3 and Figure 8C). The inclusion of the weights of the desirability approach in the optimization problem, attacked by the gridded method, as described in the *Simplex method* section, is shown to limit effectively the impact of weight selection on the identification of optimal conditions in the presence of multiple responses. In all three case studies, this led to the identification of optima (□ and ▽ in Figures 1, 2, 3, 4) which corresponded to Pareto set members offering a balanced and good performance across all three responses and which were consistent with the behavior of the given system.

The inclusion of the weights as inputs in the optimization problem leads to an increase of dimensionality. Despite this, a significant number of Simplex searches identified conditions with improved performance across the three responses, even after a small number of evaluations (Figure 7). Likewise, 24 evaluations were adequate for more than 90% of the Simplex searches to reach to a condition with a *D*_*T*_ value within 1% of the one at xw_*_ for Case Study 1 (Figure 7A). These increased to 28 and 25 for Case Studies 2 and 3, respectively (data not shown). If the Simplex method was, however, to be deployed until 15 conditions had been evaluated (including the seven conditions required for initializing a search), then a small number of searches would return such favorable conditions and a significant number (at least 60%) would lead to conditions with *D*_*T*_ values within 5% of the maximum (Figure 7). As the deployment of a composite design in three factors would require 15 unique points, it could be argued that the Simplex‐based approach is less efficient than a response surface design approach. This would need to consider that the built regression models would be of second order and hence, most likely, be less capable of accounting for the observed data trends. As indicated by the Supporting Information, and the mesh plots in Figures 1, 2, 3, these are highly nonlinear as the calibrated models include numerous terms of 3rd and 4th order. Conversely, the deployment of the Simplex method required, on average (±1 standard deviation), 27 ± 8, 28 ± 10, and 23 ± 8 evaluations to reach to the global scalar optimum for Case Studies 1–3 respectively. Hence, this approach is successful for the implementation of rapid data‐driven multi‐objective optimization via the grid compatible Simplex method.

#### Grid Compatible Simplex Method vs. Regression Analysis‐Based DoE Approach

The aforementioned complexity of the data trends in the case studies, for the considered responses, presented a severe challenge to the regression analysis DoE‐based approach despite of the adoption of the highest order models supported by the available experimental data. Even considering the best performance of the DoE approach alone, obtained when more lenient success criteria were in place, was not adequate for establishing it as the more successful approach between the two for the challenging case studies used in this work. This holds even when only the convergence to the global scalar optimum is considered for the Simplex method (i.e., success rates of 63.7, 92.3, and 48.3% vs. 66.67, 92.59, and 0% for Case Studies 1–3 respectively). Taking also into account the cases where a near optimal condition was reached by the Simplex method enhances its superiority over the approach based on the regression models for all case studies.

The unsuccessful application of the high‐order regression models for Case Study 3 serves to highlight an additional and important feature; the experimental effort made when adopting a modelling approach, may not always be justified by the returned results. In contrast, the Simplex method will always reach to a solution even if this is not the global optimum. In Case Study 3, for example, relying on the model predictions would be wasteful for process development since this approach was entirely unsuccessful based on the used criteria (Figure 8C). The deployment of the Simplex method, conversely, reached either the global scalar optimum or a local optimum, representing a viable alternative operating point, by selecting for evaluation only a subset of the conditions in the search space (*Identification of optima* section). Such results provide additional corroboration for the conclusions drawn from previous investigations regarding the difficulties met by DoE‐based approaches in the face of complex data sets.^11^, ^12^

#### Implementation of Scouting Studies Via the Grid Compatible Simplex Method

While the gridded Simplex method has been shown to be effective, efficient, and versatile for numerous case studies, its implementation is hampered by the lack of a stand‐alone software package that would make it readily accessible to end‐users. Currently, the method requires the interaction with general computing software which necessitates programming competence. At the same time, while the Simplex method is well known and established (e.g., Design Expert uses it for optimization purposes), the same may not necessarily apply for experimenters who are the intended users. The presence of a software would assist greatly to overcome these; it would allow the technique to be deployed as a functional efficient algorithm requiring only an initial method set‐up to return results since the method does not require any data analysis steps and computations are performed automatically and fast (sub‐minute). This would cover aspects of the method including problem definition (e.g., grid set up, multi‐objective, and single objective function optimization, etc.) and initialization (i.e., where to start a Simplex search from). This format is similar to optimization‐based machine learning approaches which can adopt derivative‐free population optimization techniques. While these might be more capable of identifying global optima than Simplex‐based techniques, they are not as efficient. This limitation has led to the combination of both optimization methods in numerical optimization applications (e.g., Refs. 31, 32). Machine learning applications can also be model based through the deployment of sophisticated regression methods such as artificial neural networks and support vector regression.^33^ While such techniques can potentially cope better with complex data sets, they require rigorous and expert model building, including calibration, verification, and validation, which can render them expensive to use during typical HT studies in early bioprocess development like those presented here. Hence, the Simplex method presents an attractive approach for challenging HT case studies where even high‐order regression models have limited success and other more sophisticated methods are expensive. However, the lack of an easily accessible software can make its deployment in case studies where the trends are expected to be linear and simple to be less worthwhile compared to a typical DoE approach.

Finally, as mentioned in the *Grid compatible Simplex algorithm* section, the Simplex method was deployed here to simulate its intended contemporaneous deployment with data generation. In its intended deployment, the method would be used in these case studies as a support for analytics, as described in Ref. 11, since the studies made use of slurry plates. In other cases, such as studies using miniature columns and other HT techniques with low parallelization levels, the method can be used to guide, at the same time, both the selection of conditions to be evaluated and their analysis. The fast computational execution of the method is essential in this as the involved computations in each iteration are radically faster than the receipt of analytical results and hence they do not pose a rate limiting step in process development activities. In such situations, the ability of the Simplex method to also deliver the demonstrated large performance improvements in a small number of tests, even in complex search spaces like those investigated here, would further enhance its capability to assist in challenging early stage development problems compared to other approaches. The *in vitro* contemporaneous deployment of the Simplex method to challenging early bioprocess development situations, and its benefits, will be demonstrated in future work.

## Conclusions

The identification of process feasible windows of operation can require the concurrent consideration of multiple performance metrics for a given system. However, the traditional approach of overlaying response surfaces graphically can become challenged in the presence of multiple responses. In such cases, an alternative approach can be adopted wherein the responses are combined in a composite objective function. Here, the desirability approach was used in three case studies in HT chromatography to investigate simultaneously the effects of *pH*, *Conductivity*, and *Load* on outputs including yield, and HCP and host cell DNA clearance. Obtaining optima through a regression analysis‐based DoE approach was found to have limited success; even high‐order regression models failed to return both optimal conditions and account for the data trends in the obtained desirabilities. By contrast, the deployment of the grid compatible Simplex method was significantly more successful in terms of efficacy and efficiency. Since the deterministic specification of response weights in the desirability approach may lead to conditions which fail to reflect the performance of the investigated unit operation, the weights were made part of the optimization problem. Hence, optimal conditions in terms of inputs and weights were returned by the Simplex method rapidly. This resulted in the determination of operating conditions offering a balanced performance across all outputs and it facilitated the implementation of the grid compatible Simplex method in multi‐objective optimization problems. The presented results provide further evidence of the suitability of the Simplex method to early stage bioprocess development activities.

## Conflict of Interest

The authors declare no financial or commercial conflict of interest.

**Nomenclature**
*D*: total desirability
*D*_*i*_: total desirability for *i*th set of weights
$\hat{D_{i}}$: predicted total desirability for *i*th set of weights
*d*_*k*_: individual desirability of *k*th response
*D*_*T*_: total desirability across all *I* sets of weights
*i*: *i*th set of weights and row in matrix of weights
*k*: *k*th response
*K*: number of responses
*L*_*k*_: lower limit of *k*th response
$r_{D_{i},\hat{D_{i}}}$: Pearson correlation coefficient between measured and predicted total desirability for *i*th set of weights
*T*_*k*_: target value of *k*th response
*U*_*k*_: upper limit of *k*th response
*w*_*k*_: weight for *k* ^*th*^ response
*X*: space comprised of experimental conditions
*x*_*i*, *_: grid location with maximum total desirability for *i*th set of weights
$\hat{x_{i,*}}$: predicted grid location with maximum total desirability for *i*th set of weights
*XW*: space comprised of experimental conditions and weights
xw_*_: experimental condition and weights leading to maximal total desirability across all *I* sets of weights
*y*_*k*_: *k*th response
$\hat{y_{k}}$: predicted *k*th response
*ρ*: Spearman's rank correlation coefficient

**Abbreviations**
HCP: Host cell protein.
HT: High throughput.
pI: isoelectric point.

## Supporting information


**Appendix S1**: Supporting Information
