# Supplementary material for: Data‐driven multi‐objective optimization via grid compatible simplex technique and desirability approach for challenging high throughput chromatography applications
Source: Biotechnol Prog. 2018 Oct 9;34(6):1393–406. doi: 10.1002/btpr.2673 (PMC6585819; doi:10.1002/btpr.2673)
Supplement: Supplementary file 1 — Appendix S1: Supporting Information [file BTPR-34-1393-s001.docx]

**SUPPORTING INFORMATION**

**S1: Expanded description of performance metrics for model based approach:**

The assignment of monotonically increasing integers to the levels of each factor in *X* (Section 2.2) generates hypercubes with a unit edge length. Then, two vertices for which $\lambda_{x_{i*},\hat{x}_{i*}}$=1 are adjacent to each other whereas when $\lambda_{x_{i*},\hat{x}_{i*}}$=$\sqrt{2}$ the two vertices will lie on the same 2–face. Within the context of DoE, and the analysis presented here, if a model missed the optimum $x_{i*}$, the likelihood of including it within the search space of a follow–up study increases as $\lambda_{x_{i*},\hat{x}_{i*}}$ decreases from $\sqrt{2}$. Hence, the limiting values employed for $r_{D_{i},\hat{D}_{i}}$ ($\sqrt{0.60}$ and $\sqrt{0.95}$) and $\lambda_{x_{i*},\hat{x}_{i*}}$(1 and$\sqrt{2}$) were chosen to distinguish between situations where a model accounted for trends and pinpointed optima successfully ($r_{D_{i},\hat{D}_{i}}\geq\sqrt{0.95}$ and $\lambda_{x_{i*},\hat{x}_{i*}}\leq1$), with a limited success ($\sqrt{0.95}>r_{D_{i},\hat{D}_{i}}\geq\sqrt{0.60}$ and $1<\lambda_{x_{i*},\hat{x}_{i*}}\leq\sqrt{2}$), and in all other cases unsuccessfully. Finally, a success rate of the regression analysis approach per case study was defined to be the number of cases wherein the success criteria were met over the total number of cases (i.e., 27 per case study, one for each set of weights). This represented the general suitability of a regression analysis approach for a given case study.

**S2: Application of Simplex method in multi-objective optimization applications – Expansion of input space and an executed example**

The proposed methodology includes the weights of each of the considered responses in the independent variables of the optimization problem. This requires the expansion of the input space, *X*, to a space *XW* by the inclusion of the weights, *W*, of the desirability approach as shown graphically in Scheme 1. Then, the objective function to be optimized is comprised of the amalgamated responses across both the entire input space *X* and the weights *W*. In Scheme 1, the input space *X* includes 48 unique grid locations in a three dimensional space (*pH*, *Load*, and *Conductivity*). The weights *W* are comprised of 27 unique triplets of weights on the individual three responses (*Yield*, *HCP content*, and *DNA content*). Hence, the objective function, *D_T_*, is comprised of 27 sets of average total desirabilities, $\bar{D}$, each with 48 members (i.e., *D_T_* has a total of 48×27 = 1296 values), calculated by combining the three responses across the input space *X* in 27 different ways. An executed example is provided next, but first it needs to be clarified that in the intended contemporaneous deployment of the Simplex method, the objective function *D_T_* would not be available as shown in Scheme 1; instead it would be determined gradually as the Simplex method would suggest experimental conditions to be evaluated and weights to be adopted for the amalgamation of the corresponding and measured multiple responses.

In the provided example, it is assumed that a gridded space *X* is created by considering two inputs, or factors, *pH* and *[Salt]* in a HT investigation of binding conditions for a chromatography capture step wherein conditions are screened to maximize *Yield* and *Purity*. It is assumed that the space is created by the combination of two *pH* levels (e.g., 5 and 6) and four *[Salt]* levels (e.g., 50 mM, 100 mM, 150 mM, and 200 mM). Furthermore, in this example, since both *Yield* and *Purity* are maximized (i.e., Equation 1), the target value, *T*, is set at 100% for both responses and the lower limit, *L*, is set at 40% and 50% respectively. Finally, the achieved yields and purities for each of the 8 conditions in this space are combined through the desirability approach by employing two weights for *Yield* (e.g., *w_y_* equal to 1 and 2) and three weights for *Purity* (e.g., *w_p_* equal to 0.5, 1 and 1.5) leading to 6 weight combinations. The data are tabulated in Table S1. This also includes the made calculations for the individual desirabilities for the two responses (Equation 1) and the total desirability (Equation 3).

| 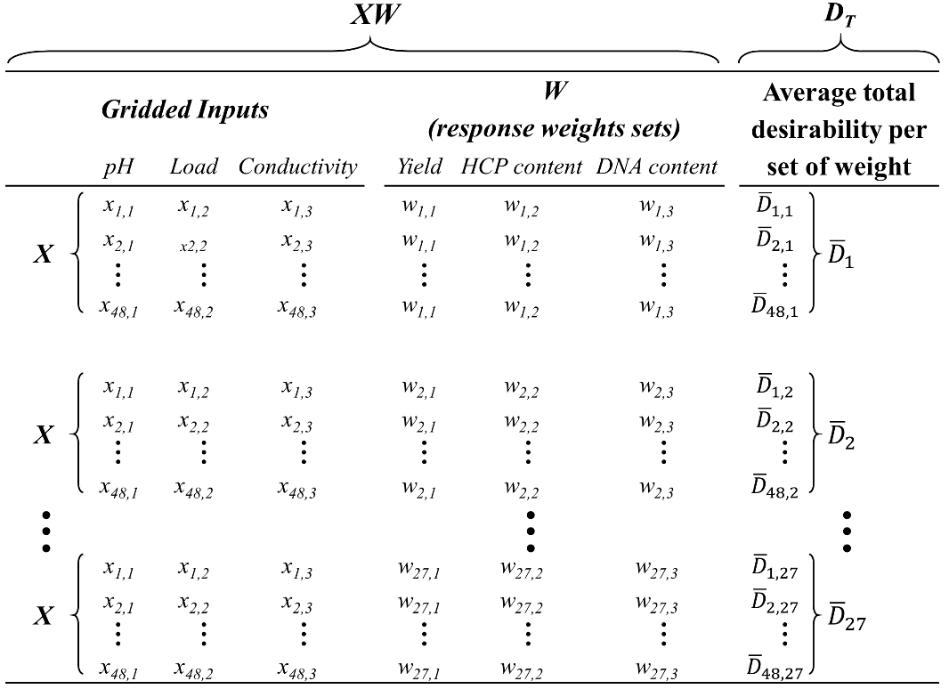 | **Scheme 1** |
| --- | --- |

As Table S1 indicates the combination of the weights and conditions in the input space returns the space *XW* which is comprised of 48 conditions. Each of these conditions is a coordinate of the experimental conditions in *pH* and *[Salt]* and the weights of the *Yield* (*w_y_*) and *Purity* (*w_p_*) responses. Consequently, each of these conditions is also associated to a total desirability value (i.e., *D*) which are joined in a new response, *D_T_*. The deployment of the Simplex method in the *XW* space would therefore aim to maximize *D_T_* and hence identify the second condition in *XW* (Table S1). As mentioned previously, in the intended deployment of the Simplex approach, the response *D_T_* would not be entirely known to the experimenter; instead the method would suggest, iteratively, conditions to be tested in the *XW* space until the method converged to the optimum (i.e., the condition leading to the maximum of *D_T_*).

**Table S1: Data of executed example including the input space *X*, the responses *Yield* and *Purity*, the weights of the responses in the weights space *W*, and the calculated individual (*d_yield_* and *d_purity_*) and total desirabilities (*D*) for each condition in the input space and weight on the responses.**

| **Condition in *XW*** | **Condition in *X*** | **Weight combination** | ***XW* space** | | | | **Responses** | | **Individual desirabilities** | | ***D_T_* obj. function** |
| --- | --- | --- | --- | --- | --- | --- | --- | --- | --- | --- | --- |
|  |  |  | ***X* space** | | ***W* space** | |  |  |  |  |  |
|  |  |  | *pH* | *[Salt]* | *w_y_* | *w_p_* | *%Yield* | *%Purity* | *d_yield_* | *d_purity_* | *D (total desirability)* |
| 1 | 1 | 1 | 5 | 50 | 1 | 0.5 | 80 | 90 | 0.67 | 0.89 | 0.77 |
| **2** | **2** | **1** | **5** | **100** | **1** | **0.5** | **85** | **85** | **0.75** | **0.84** | **0.79** |
| 3 | 3 | 1 | 5 | 150 | 1 | 0.5 | 70 | 65 | 0.50 | 0.55 | 0.52 |
| 4 | 4 | 1 | 5 | 200 | 1 | 0.5 | 60 | 50 | 0.33 | 0.00 | 0.00 |
| 5 | 5 | 1 | 6 | 50 | 1 | 0.5 | 70 | 60 | 0.50 | 0.45 | 0.47 |
| 6 | 6 | 1 | 6 | 100 | 1 | 0.5 | 62 | 50 | 0.37 | 0.00 | 0.00 |
| 7 | 7 | 1 | 6 | 150 | 1 | 0.5 | 50 | 40 | 0.17 | 0.00 | 0.00 |
| 8 | 8 | 1 | 6 | 200 | 1 | 0.5 | 30 | 30 | 0.00 | 0.00 | 0.00 |
| 9 | 1 | 2 | 5 | 50 | 1 | 1.0 | 80 | 90 | 0.67 | 0.80 | 0.73 |
| 10 | 2 | 2 | 5 | 100 | 1 | 1.0 | 85 | 85 | 0.75 | 0.70 | 0.72 |
| 11 | 3 | 2 | 5 | 150 | 1 | 1.0 | 70 | 65 | 0.50 | 0.30 | 0.39 |
| 12 | 4 | 2 | 5 | 200 | 1 | 1.0 | 60 | 50 | 0.33 | 0.00 | 0.00 |
| 13 | 5 | 2 | 6 | 50 | 1 | 1.0 | 70 | 60 | 0.50 | 0.20 | 0.32 |
| 14 | 6 | 2 | 6 | 100 | 1 | 1.0 | 62 | 50 | 0.37 | 0.00 | 0.00 |
| 15 | 7 | 2 | 6 | 150 | 1 | 1.0 | 50 | 40 | 0.17 | 0.00 | 0.00 |
| 16 | 8 | 2 | 6 | 200 | 1 | 1.0 | 30 | 30 | 0.00 | 0.00 | 0.00 |
| 17 | 1 | 3 | 5 | 50 | 1 | 1.5 | 80 | 90 | 0.67 | 0.72 | 0.69 |
| 18 | 2 | 3 | 5 | 100 | 1 | 1.5 | 85 | 85 | 0.75 | 0.59 | 0.66 |
| 19 | 3 | 3 | 5 | 150 | 1 | 1.5 | 70 | 65 | 0.50 | 0.16 | 0.29 |
| 20 | 4 | 3 | 5 | 200 | 1 | 1.5 | 60 | 50 | 0.33 | 0.00 | 0.00 |
| 21 | 5 | 3 | 6 | 50 | 1 | 1.5 | 70 | 60 | 0.50 | 0.09 | 0.21 |
| 22 | 6 | 3 | 6 | 100 | 1 | 1.5 | 62 | 50 | 0.37 | 0.00 | 0.00 |
| 23 | 7 | 3 | 6 | 150 | 1 | 1.5 | 50 | 40 | 0.17 | 0.00 | 0.00 |
| 24 | 8 | 3 | 6 | 200 | 1 | 1.5 | 30 | 30 | 0.00 | 0.00 | 0.00 |
| 25 | 1 | 4 | 5 | 50 | 2 | 0.5 | 80 | 90 | 0.44 | 0.89 | 0.63 |
| 26 | 2 | 4 | 5 | 100 | 2 | 0.5 | 85 | 85 | 0.56 | 0.84 | 0.69 |
| 27 | 3 | 4 | 5 | 150 | 2 | 0.5 | 70 | 65 | 0.25 | 0.55 | 0.37 |
| 28 | 4 | 4 | 5 | 200 | 2 | 0.5 | 60 | 50 | 0.11 | 0.00 | 0.00 |
| 29 | 5 | 4 | 6 | 50 | 2 | 0.5 | 70 | 60 | 0.25 | 0.45 | 0.33 |
| 30 | 6 | 4 | 6 | 100 | 2 | 0.5 | 62 | 50 | 0.13 | 0.00 | 0.00 |
| 31 | 7 | 4 | 6 | 150 | 2 | 0.5 | 50 | 40 | 0.03 | 0.00 | 0.00 |
| 32 | 8 | 4 | 6 | 200 | 2 | 0.5 | 30 | 30 | 0.00 | 0.00 | 0.00 |
| 33 | 1 | 5 | 5 | 50 | 2 | 1.0 | 80 | 90 | 0.44 | 0.80 | 0.60 |
| 34 | 2 | 5 | 5 | 100 | 2 | 1.0 | 85 | 85 | 0.56 | 0.70 | 0.63 |
| 35 | 3 | 5 | 5 | 150 | 2 | 1.0 | 70 | 65 | 0.25 | 0.30 | 0.27 |
| 36 | 4 | 5 | 5 | 200 | 2 | 1.0 | 60 | 50 | 0.11 | 0.00 | 0.00 |
| 37 | 5 | 5 | 6 | 50 | 2 | 1.0 | 70 | 60 | 0.25 | 0.20 | 0.22 |
| 38 | 6 | 5 | 6 | 100 | 2 | 1.0 | 62 | 50 | 0.13 | 0.00 | 0.00 |
| 39 | 7 | 5 | 6 | 150 | 2 | 1.0 | 50 | 40 | 0.03 | 0.00 | 0.00 |
| 40 | 8 | 5 | 6 | 200 | 2 | 1.0 | 30 | 30 | 0.00 | 0.00 | 0.00 |
| 41 | 1 | 6 | 5 | 50 | 2 | 1.5 | 80 | 90 | 0.44 | 0.72 | 0.56 |
| 42 | 2 | 6 | 5 | 100 | 2 | 1.5 | 85 | 85 | 0.56 | 0.59 | 0.57 |
| 43 | 3 | 6 | 5 | 150 | 2 | 1.5 | 70 | 65 | 0.25 | 0.16 | 0.20 |
| 44 | 4 | 6 | 5 | 200 | 2 | 1.5 | 60 | 50 | 0.11 | 0.00 | 0.00 |
| 45 | 5 | 6 | 6 | 50 | 2 | 1.5 | 70 | 60 | 0.25 | 0.09 | 0.15 |
| 46 | 6 | 6 | 6 | 100 | 2 | 1.5 | 62 | 50 | 0.13 | 0.00 | 0.00 |
| 47 | 7 | 6 | 6 | 150 | 2 | 1.5 | 50 | 40 | 0.03 | 0.00 | 0.00 |
| 48 | 8 | 6 | 6 | 200 | 2 | 1.5 | 30 | 30 | 0.00 | 0.00 | 0.00 |

**S3: Mesh plots of *D_T_*values per case study.**


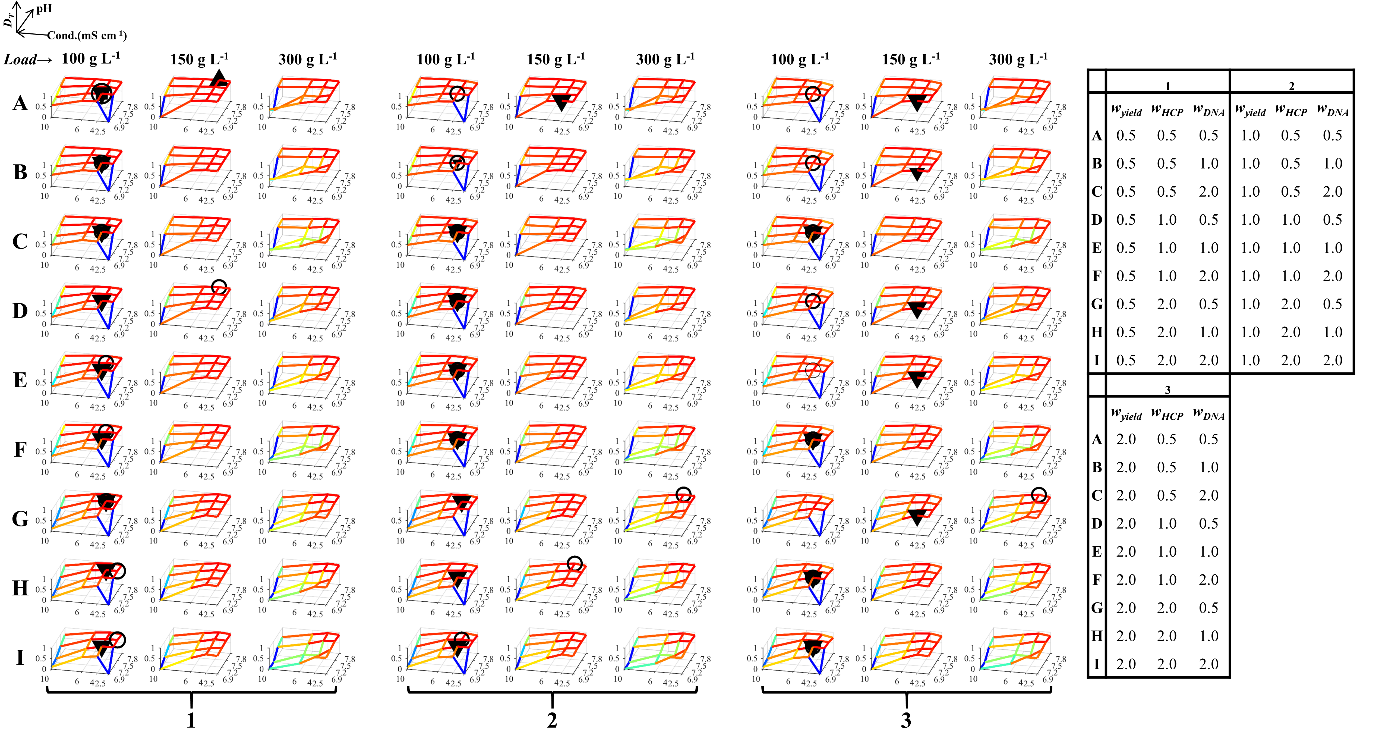


Figure S1: Mesh plot of concatenated averaged measured total desirabilities, *D_T_*, for Case Study 1 across the twenty seven sets of weights. Each row of plots, (A) – (I), corresponds to a different set of weights for the HCP and DNA content responses whereas the numbers at the bottom of the plots indicate different weights for the yield response. These are detailed in the table at the right hand side of the figure. (○) total desirability optima per set of weights based on the averaged raw measurements; (■) global scalar optimum (also Simplex–derived best condition); (▲) Simplex–derived local optimum; (▼) predicted optima per set of weights returned by the regression–based analysis approach. Blue color denotes low total desirability and red color denotes a high total desirability.


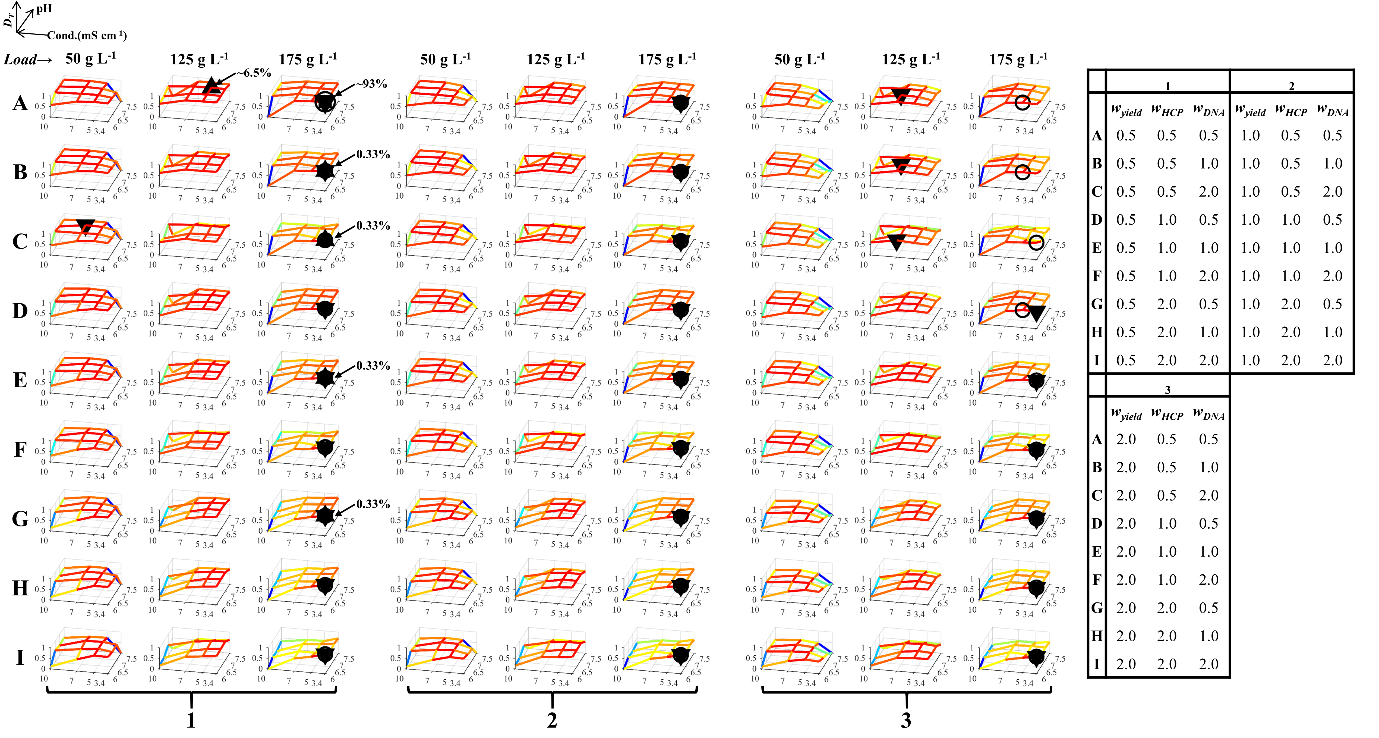


Figure S2: Mesh plot of concatenated averaged measured total desirabilities, *D_T_*, for Case Study 2 acres the twenty seven sets of weights. Each row of plots, (A) – (I), corresponds to a different set of weights for the HCP and DNA content responses whereas the numbers at the bottom of the plots indicate different weights for the yield response. These are detailed in the table at the right hand side of the figure. (○) total desirability optima per set of weights based on the averaged raw measurements; (■) global scalar optimum (also Simplex–derived best condition); (▲) Simplex–derived local optimum; (▼) predicted optima per set of weights returned by the regression–based analysis approach. Blue color denotes low total desirability and red color denotes a high total desirability.


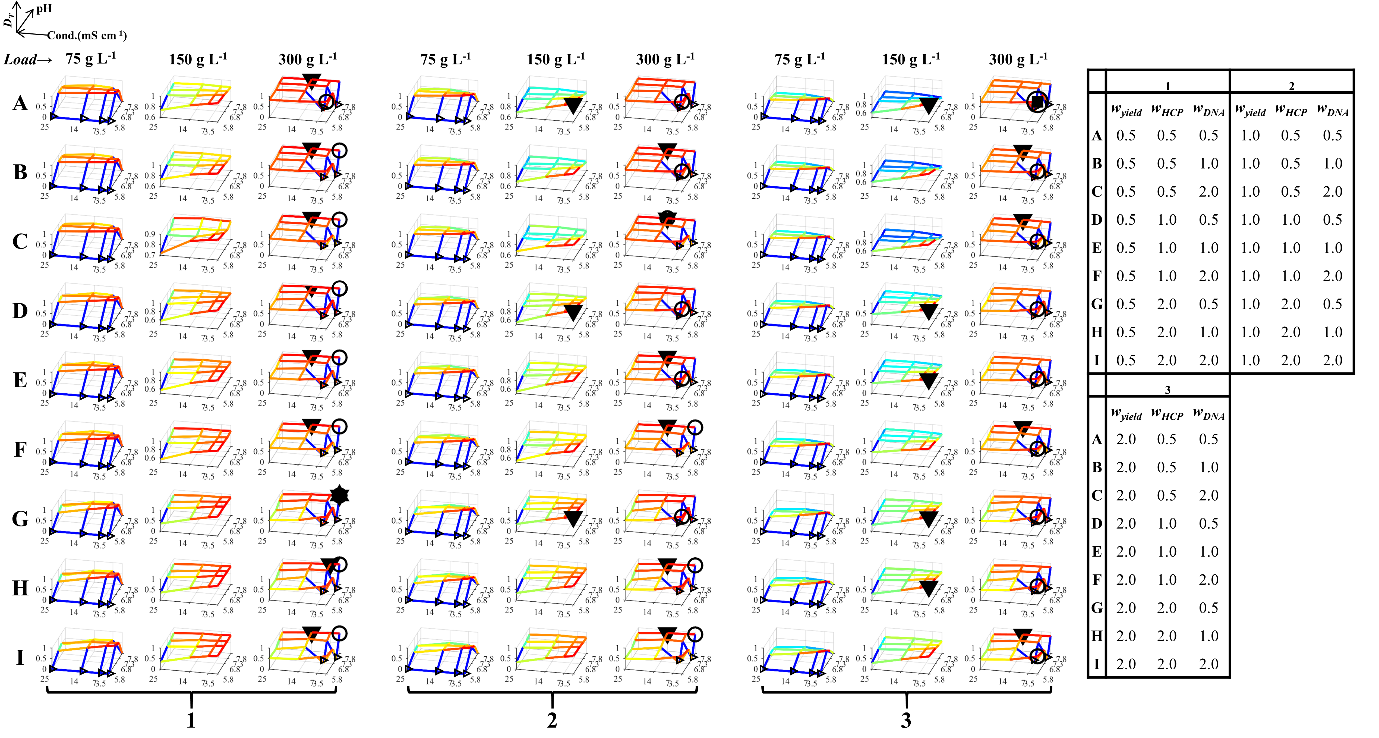


Figure S3: Mesh plot of concatenated averaged measured total desirabilities, *D_T_*, for Case Study 3 across the twenty seven sets of weights. Each row of plots, (A) – (I), corresponds to a different set of weights for the HCP and DNA content responses whereas the numbers at the bottom of the plots indicate different weights for the yield response. These are detailed in the table at the right hand side of the figure. (○) total desirability optima per set of weights based on the averaged raw measurements; (■) global scalar optimum (also Simplex–derived best condition); (▲) Simplex–derived local optimum; (▼) predicted optima per set of weights returned by the regression–based analysis approach; (▷) missing data points (replaced by a surrogate). Blue color denotes low total desirability and red color denotes a high total desirability.

**S3: Regression models for responses yield, HCP content and DNA content in Case Studies 1 – 3 and mesh plots of the resulting predicted total desirabilities in the *XW* space per set of weights.**

**High order regression model details for Case Study 1 and the three considered responses.**

|  | **Yield** | | | **HCP content** | | | **DNA content** | | |
| --- | --- | --- | --- | --- | --- | --- | --- | --- | --- |
| **%R^2^** | 77.87 | | | 99.57 | | | 86.41 | | |
| **SSE*^a^*** | 360.78 | | | 130.16 | | | 20.22 | | |
| **DFR*^b^*** | 26.00 | | | 21.00 | | | 16.00 | | |
| **DFE*^c^*** | 69.00 | | | 74.00 | | | 79.00 | | |
| **Coefficient** | **Value** | **t-value** | **p-value** | **Value** | **t-value** | **p-value** | **Value** | **t-value** | **p-value** |
| *Constant* | -2.16E+04 | -4.00E+00 | 1.55E-04 | -1.80E+03 | -2.06E+00 | 4.25E-02 | 1.59E+04 | 4.53E+00 | 2.05E-05 |
| *A: pH* | 6.93E+03 | 3.24E+00 | 1.87E-03 | 5.47E+02 | 2.29E+00 | 2.51E-02 | -6.50E+03 | -4.53E+00 | 2.07E-05 |
| *B: Load^d^* | 8.97E+01 | 5.16E+00 | 2.29E-06 | 1.57E+01 | 2.39E+00 | 1.92E-02 | -8.23E+01 | -6.19E+00 | 2.53E-08 |
| *C: Cond.^e^* | 1.80E+03 | 5.43E+00 | 8.02E-07 | -3.70E+03 | -7.68E+00 | 5.20E-11 | -8.06E+02 | -2.01E+00 | 4.83E-02 |
| *A^2^* | -7.03E+02 | -2.46E+00 | 1.65E-02 | -4.07E+01 | -2.48E+00 | 1.54E-02 | 8.84E+02 | 4.53E+00 | 2.09E-05 |
| *A×B* | -2.33E+01 | -4.92E+00 | 5.73E-06 | -3.27E+00 | -2.44E+00 | 1.69E-02 | 3.37E+01 | 6.20E+00 | 2.44E-08 |
| *B^2^* | -1.71E-01 | -4.15E+00 | 9.39E-05 | -5.54E-02 | -2.34E+00 | 2.22E-02 | p-value>0.1 | | |
| *A×C* | -4.35E+02 | -4.91E+00 | 5.82E-06 | 1.50E+03 | 7.63E+00 | 6.58E-11 | 3.35E+02 | 2.04E+00 | 4.43E-02 |
| *B×C* | -3.64E+00 | -5.68E+00 | 3.00E-07 | p-value>0.1 | | | -3.06E-01 | -2.37E+00 | 2.03E-02 |
| *C^2^* | -1.12E+02 | -4.16E+00 | 8.95E-05 | p-value>0.1 | | | p-value>0.1 | | |
| *A^3^* | 2.18E+01 | 1.69E+00 | 9.47E-02 | p-value>0.1 | | | -4.01E+01 | -4.52E+00 | 2.12E-05 |
| *A^2^×B* | 1.52E+00 | 4.72E+00 | 1.20E-05 | p-value>0.1 | | | -4.59E+00 | -6.20E+00 | 2.37E-08 |
| *A×B^2^* | 4.43E-02 | 3.95E+00 | 1.87E-04 | 1.56E-02 | 2.41E+00 | 1.83E-02 | p-value>0.1 | | |
| *A^2^×C* | 2.67E+01 | 4.45E+00 | 3.16E-05 | -2.06E+02 | -7.72E+00 | 4.41E-11 | -4.65E+01 | -2.08E+00 | 4.06E-02 |
| *A×B×C* | 7.69E-01 | 4.71E+00 | 1.22E-05 | p-value>0.1 | | | 8.50E-02 | 2.41E+00 | 1.82E-02 |
| *B^2^×C* | 2.09E-03 | 3.77E+00 | 3.41E-04 | -3.54E-04 | -3.37E+00 | 1.20E-03 | p-value>0.1 | | |
| *A×C^2^* | 2.38E+01 | 3.58E+00 | 6.43E-04 | 5.03E+00 | 5.40E+00 | 7.70E-07 | p-value>0.1 | | |
| *B×C^2^* | 5.51E-02 | 4.17E+00 | 8.83E-05 | 1.42E-02 | 3.67E+00 | 4.59E-04 | -2.64E-04 | -3.38E+00 | 1.12E-03 |
| *C^3^* | 2.07E+00 | 2.84E+00 | 5.93E-03 | -1.82E+00 | -4.93E+00 | 4.80E-06 | p-value>0.1 | | |
| *A^3^×B* | p-value>0.1 | | | 2.13E-02 | 2.59E+00 | 1.15E-02 | 2.08E-01 | 6.21E+00 | 2.34E-08 |
| *A^2^×B^2^* | -2.89E-03 | -3.78E+00 | 3.27E-04 | -1.10E-03 | -2.50E+00 | 1.48E-02 | p-value>0.1 | | |
| *A^3^×C* | p-value>0.1 | | | 9.57E+00 | 7.92E+00 | 1.80E-11 | 2.15E+00 | 2.12E+00 | 3.73E-02 |
| *A^2^×B×C* | -4.27E-02 | -3.94E+00 | 1.95E-04 | -1.42E-03 | -3.58E+00 | 6.16E-04 | -5.87E-03 | -2.45E+00 | 1.67E-02 |
| *A×B^2^×C* | -2.17E-04 | -2.99E+00 | 3.88E-03 | 5.18E-05 | 3.55E+00 | 6.80E-04 | p-value>0.1 | | |
| *A^2^×C^2^* | -1.25E+00 | -2.88E+00 | 5.27E-03 | -6.63E-01 | -5.33E+00 | 1.02E-06 | p-value>0.1 | | |
| *A×B×C^2^* | -3.53E-03 | -2.57E+00 | 1.25E-02 | p-value>0.1 | | | p-value>0.1 | | |
| *B^2^×C^2^* | -3.27E-05 | -2.83E+00 | 6.03E-03 | p-value>0.1 | | | 6.78E-07 | 2.66E+00 | 9.54E-03 |
| *A×C^3^* | -2.52E-01 | -2.55E+00 | 1.30E-02 | 2.46E-01 | 4.92E+00 | 5.12E-06 | p-value>0.1 | | |
| *B×C^3^* | -7.85E-04 | -2.01E+00 | 4.80E-02 | -8.61E-04 | -4.03E+00 | 1.33E-04 | p-value>0.1 | | |

*a*, sum of squared errors; *b*, degrees of freedom of regression model; *c*, degrees of freedom of residuals; *d*, *Load* (g L^-1^); *e*, *Conductivity* (mS cm^-1^).

**High order regression model details for Case Study 2 and the three considered responses.**

|  | **Yield** | | | **HCP content** | | | **DNA content** | | |
| --- | --- | --- | --- | --- | --- | --- | --- | --- | --- |
| **%R^2^** | 77.64 | | | 95.46 | | | 74.99 | | |
| **SSE*^a^*** | 124.06 | | | 43212.06 | | | 1001.15 | | |
| **DFR*^b^*** | 10.00 | | | 12.00 | | | 18.00 | | |
| **DFE*^c^*** | 85.00 | | | 83.00 | | | 77.00 | | |
| **Coefficient** | **Value** | **t-value** | **p-value** | **Value** | **t-value** | **p-value** | **Value** | **t-value** | **p-value** |
| *Constant* | 1.02E+02 | 4.22E+01 | 7.85E-59 | 1.19E+02 | 1.33E+00 | 1.86E-01 | -6.46E+03 | -4.72E+00 | 1.02E-05 |
| *A: pH* | p-value>0.1 | | | p-value>0.1 | | | 2.76E+03 | 4.41E+00 | 3.29E-05 |
| *B: Load^d^* | 5.30E-02 | 3.68E+00 | 4.12E-04 | p-value>0.1 | | | 1.08E+01 | 3.64E+00 | 4.85E-04 |
| *C: Cond.^e^* | p-value>0.1 | | | 2.84E+03 | 2.27E+00 | 2.57E-02 | 3.47E+01 | 3.21E+00 | 1.91E-03 |
| *A^2^* | -5.10E-01 | -6.61E+00 | 3.18E-09 | p-value>0.1 | | | -3.91E+02 | -4.10E+00 | 1.03E-04 |
| *A×B* | p-value>0.1 | | | p-value>0.1 | | | p-value>0.1 | | |
| *B^2^* | p-value>0.1 | | | p-value>0.1 | | | -1.47E-01 | -3.89E+00 | 2.12E-04 |
| *A×C* | p-value>0.1 | | | -1.22E+03 | -2.19E+00 | 3.16E-02 | -5.38E+00 | -3.37E+00 | 1.16E-03 |
| *B×C* | -1.18E-01 | -4.00E+00 | 1.33E-04 | -4.83E+00 | -4.00E+00 | 1.35E-04 | -7.84E-01 | -3.26E+00 | 1.69E-03 |
| *C^2^* | -1.35E-01 | -3.54E+00 | 6.43E-04 | 4.31E+01 | 2.47E+00 | 1.57E-02 | p-value>0.1 | | |
| *A^3^* | p-value>0.1 | | | p-value>0.1 | | | 1.86E+01 | 3.80E+00 | 2.83E-04 |
| *A^2^×B* | p-value>0.1 | | | p-value>0.1 | | | -6.67E-01 | -3.55E+00 | 6.56E-04 |
| *A×B^2^* | p-value>0.1 | | | p-value>0.1 | | | 4.14E-02 | 3.72E+00 | 3.72E-04 |
| *A^2^×C* | 5.69E-02 | 5.05E+00 | 2.52E-06 | 1.69E+02 | 2.04E+00 | 4.45E-02 | p-value>0.1 | | |
| *A×B×C* | 2.60E-02 | 3.31E+00 | 1.38E-03 | 1.45E+00 | 4.03E+00 | 1.24E-04 | 1.31E-01 | 3.85E+00 | 2.46E-04 |
| *B^2^×C* | p-value>0.1 | | | p-value>0.1 | | | 4.28E-03 | 4.04E+00 | 1.26E-04 |
| *A×C^2^* | p-value>0.1 | | | -5.71E+00 | -2.46E+00 | 1.61E-02 | p-value>0.1 | | |
| *B×C^2^* | 7.84E-03 | 4.91E+00 | 4.29E-06 | p-value>0.1 | | | -1.68E-02 | -1.70E+00 | 9.41E-02 |
| *C^3^* | p-value>0.1 | | | -1.98E+00 | -1.76E+00 | 8.24E-02 | p-value>0.1 | | |
| *A^3^×B* | p-value>0.1 | | | p-value>0.1 | | | 6.21E-02 | 3.40E+00 | 1.05E-03 |
| *A^2^×B^2^* | p-value>0.1 | | | p-value>0.1 | | | -2.89E-03 | -3.53E+00 | 7.04E-04 |
| *A^3^×C* | p-value>0.1 | | | -7.56E+00 | -1.85E+00 | 6.74E-02 | p-value>0.1 | | |
| *A^2^×B×C* | -1.24E-03 | -2.17E+00 | 3.29E-02 | -1.08E-01 | -4.04E+00 | 1.19E-04 | p-value>0.1 | | |
| *A×B^2^×C* | -4.23E-06 | -2.78E+00 | 6.63E-03 | p-value>0.1 | | | -5.76E-04 | -3.78E+00 | 3.04E-04 |
| *A^2^×C^2^* | p-value>0.1 | | | p-value>0.1 | | | p-value>0.1 | | |
| *A×B×C^2^* | -1.15E-03 | -5.63E+00 | 2.24E-07 | p-value>0.1 | | | p-value>0.1 | | |
| *B^2^×C^2^* | p-value>0.1 | | | p-value>0.1 | | | -4.63E-05 | -2.54E+00 | 1.30E-02 |
| *A×C^3^* | p-value>0.1 | | | 2.79E-01 | 1.78E+00 | 7.87E-02 | p-value>0.1 | | |
| *B×C^3^* | p-value>0.1 | | | 4.14E-04 | 2.05E+00 | 4.36E-02 | 1.22E-03 | 2.55E+00 | 1.28E-02 |

*a*, sum of squared errors; *b*, degrees of freedom of regression model; *c*, degrees of freedom of residuals; *d*, *Load* (g L^-1^); *e*, *Conductivity* (mS cm^-1^).

**High order regression model details for Case Study 3 and the three considered responses.**

|  | **Yield** | | | **HCP content** | | | **DNA content** | | |
| --- | --- | --- | --- | --- | --- | --- | --- | --- | --- |
| **%R^2^** | 99.88 | | | 97.67 | | | 97.29 | | |
| **SSE*^a^*** | 22.23 | | | 7246.48 | | | 1210.17 | | |
| **DFR*^b^*** | 23 | | | 23 | | | 22 | | |
| **DFE*^c^*** | 72 | | | 64 | | | 69 | | |
| **Coefficient** | **Value** | **t-value** | **p-value** | **Value** | **t-value** | **p-value** | **Value** | **t-value** | **p-value** |
| *Constant* | -1.53E+03 | -5.72E+00 | 2.31E-07 | 5.15E+04 | 3.68E+00 | 4.78E-04 | 9.95E+03 | 4.83E+00 | 8.07E-06 |
| *A: pH* | 7.24E+02 | 6.13E+00 | 4.24E-08 | -1.94E+04 | -3.52E+00 | 8.02E-04 | -4.28E+03 | -4.67E+00 | 1.43E-05 |
| *B: Load^d^* | 5.16E+00 | 3.67E+00 | 4.59E-04 | -2.85E+02 | -3.47E+00 | 9.23E-04 | -6.82E+01 | -6.05E+00 | 6.53E-08 |
| *C: Cond.^e^* | 1.31E+01 | 3.37E+00 | 1.22E-03 | -1.44E+03 | -4.92E+00 | 6.35E-06 | p-value>0.1 | | |
| *A^2^* | -1.04E+02 | -6.01E+00 | 6.97E-08 | 2.40E+03 | 3.33E+00 | 1.43E-03 | 6.04E+02 | 4.48E+00 | 2.90E-05 |
| *A×B* | -2.27E+00 | -3.75E+00 | 3.58E-04 | 9.03E+01 | 3.15E+00 | 2.47E-03 | 2.84E+01 | 5.68E+00 | 2.99E-07 |
| *B^2^* | -1.94E-03 | -1.72E+00 | 8.89E-02 | 4.79E-01 | 4.40E+00 | 4.12E-05 | p-value>0.1 | | |
| *A×C* | -6.57E+00 | -5.75E+00 | 2.03E-07 | 6.49E+02 | 4.99E+00 | 4.89E-06 | -9.57E+00 | -4.61E+00 | 1.78E-05 |
| *B×C* | 2.03E-02 | 2.12E+00 | 3.78E-02 | p-value>0.1 | | | 6.20E-01 | 8.30E+00 | 5.70E-12 |
| *C^2^* | -3.55E-01 | -2.90E+00 | 4.95E-03 | 5.60E-01 | 1.81E+00 | 7.56E-02 | p-value>0.1 | | |
| *A^3^* | 4.64E+00 | 5.52E+00 | 5.10E-07 | -9.84E+01 | -3.11E+00 | 2.75E-03 | -2.80E+01 | -4.27E+00 | 6.23E-05 |
| *A^2^×B* | 3.18E-01 | 3.63E+00 | 5.35E-04 | -8.78E+00 | -2.68E+00 | 9.42E-03 | -3.79E+00 | -5.17E+00 | 2.21E-06 |
| *A×B^2^* | 7.76E-04 | 2.33E+00 | 2.28E-02 | -1.30E-01 | -4.36E+00 | 4.89E-05 | -9.48E-04 | -4.36E+00 | 4.44E-05 |
| *A^2^×C* | 6.46E-01 | 7.68E+00 | 6.00E-11 | -9.78E+01 | -5.12E+00 | 3.01E-06 | 2.97E+00 | 5.30E+00 | 1.34E-06 |
| *A×B×C* | 4.80E-03 | 1.99E+00 | 5.04E-02 | 1.39E-01 | 7.60E+00 | 1.62E-10 | -1.97E-01 | -1.05E+01 | 5.91E-16 |
| *B^2^×C* | -8.05E-05 | -6.44E+00 | 1.16E-08 | -2.00E-03 | -8.76E+00 | 1.47E-12 | 3.30E-04 | 3.54E+00 | 7.32E-04 |
| *A×C^2^* | 1.54E-01 | 4.26E+00 | 6.10E-05 | p-value>0.1 | | | p-value>0.1 | | |
| *B×C^2^* | -7.62E-04 | -6.42E+00 | 1.26E-08 | -4.17E-03 | -2.30E+00 | 2.46E-02 | -3.98E-03 | -3.77E+00 | 3.36E-04 |
| *C^3^* | p-value>0.1 | | | -2.14E-02 | -2.43E+00 | 1.81E-02 | 1.33E-02 | 3.18E+00 | 2.24E-03 |
| *A^3^×B* | -1.31E-02 | -3.09E+00 | 2.85E-03 | 2.41E-01 | 1.94E+00 | 5.64E-02 | 1.64E-01 | 4.58E+00 | 1.99E-05 |
| *A^2^×B^2^* | -8.33E-05 | -3.39E+00 | 1.13E-03 | 8.80E-03 | 4.31E+00 | 5.83E-05 | 1.23E-04 | 4.01E+00 | 1.51E-04 |
| *A^3^×C* | p-value>0.1 | | | 4.92E+00 | 5.29E+00 | 1.59E-06 | -2.21E-01 | -5.75E+00 | 2.26E-07 |
| *A^2^×B×C* | -1.00E-03 | -6.00E+00 | 7.15E-08 | -1.85E-02 | -9.03E+00 | 4.97E-13 | 1.52E-02 | 1.17E+01 | 5.09E-18 |
| *A×B^2^×C* | 1.07E-05 | 5.95E+00 | 9.02E-08 | 2.69E-04 | 8.49E+00 | 4.43E-12 | -4.36E-05 | -3.25E+00 | 1.77E-03 |
| *A^2^×C^2^* | -1.43E-02 | -5.37E+00 | 9.16E-07 | p-value>0.1 | | | p-value>0.1 | | |
| *A×B×C^2^* | 1.02E-04 | 5.97E+00 | 8.34E-08 | p-value>0.1 | | | 3.86E-04 | 3.01E+00 | 3.61E-03 |
| *B^2^×C^2^* | p-value>0.1 | | | 8.93E-06 | 1.96E+00 | 5.47E-02 | p-value>0.1 | | |
| *A×C^3^* | p-value>0.1 | | | 2.75E-03 | 3.25E+00 | 1.82E-03 | -2.00E-03 | -3.32E+00 | 1.42E-03 |
| *B×C^3^* | p-value>0.1 | | | p-value>0.1 | | | 3.69E-05 | 2.62E+00 | 1.09E-02 |

*a*, sum of squared errors; *b*, degrees of freedom of regression model; *c* degrees of freedom of residuals; *d*, *Load* (g L^-1^); *e*, *Conductivity* (mS cm^-1^).


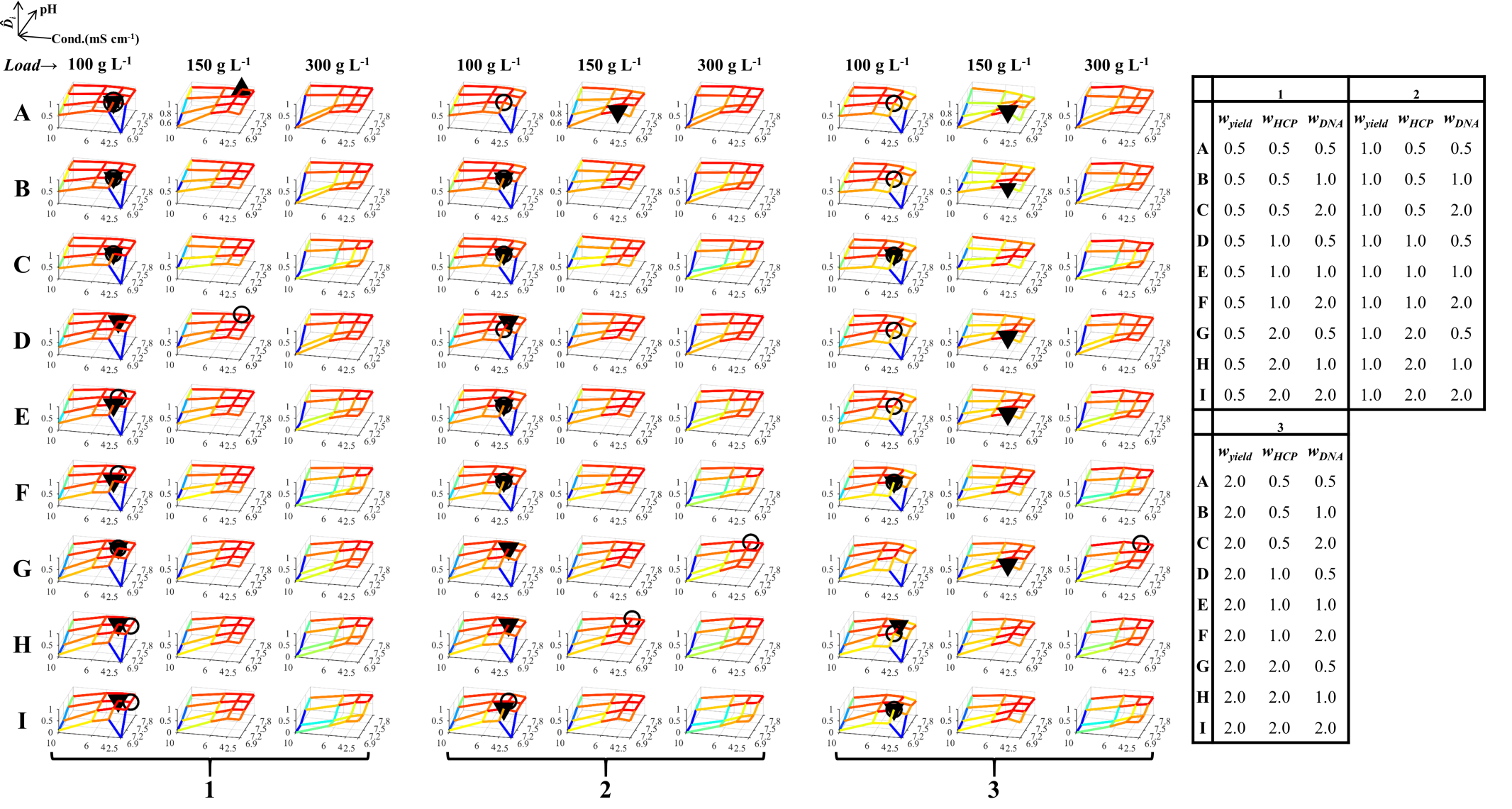


Figure S4: Mesh plot of predicted total desirability, $\hat{D}_{i}$, for Case Study 1 for the twenty seven sets of weights. Each row of plots, (A) – (I), corresponds to a different set of weights for the HCP and DNA content responses whereas the numbers at the bottom of the plots indicate different weights for the yield response. These are detailed in the table at the right hand side of the figure. (○) total desirability optima per set of weights based on the averaged raw measurements; (■) global scalar optimum (also Simplex–derived best condition); (▲) Simplex–derived local optimum; (▼) predicted optima per set of weights returned by the regression–based analysis approach. Blue color denotes low total desirability and red color denotes a high total desirability.


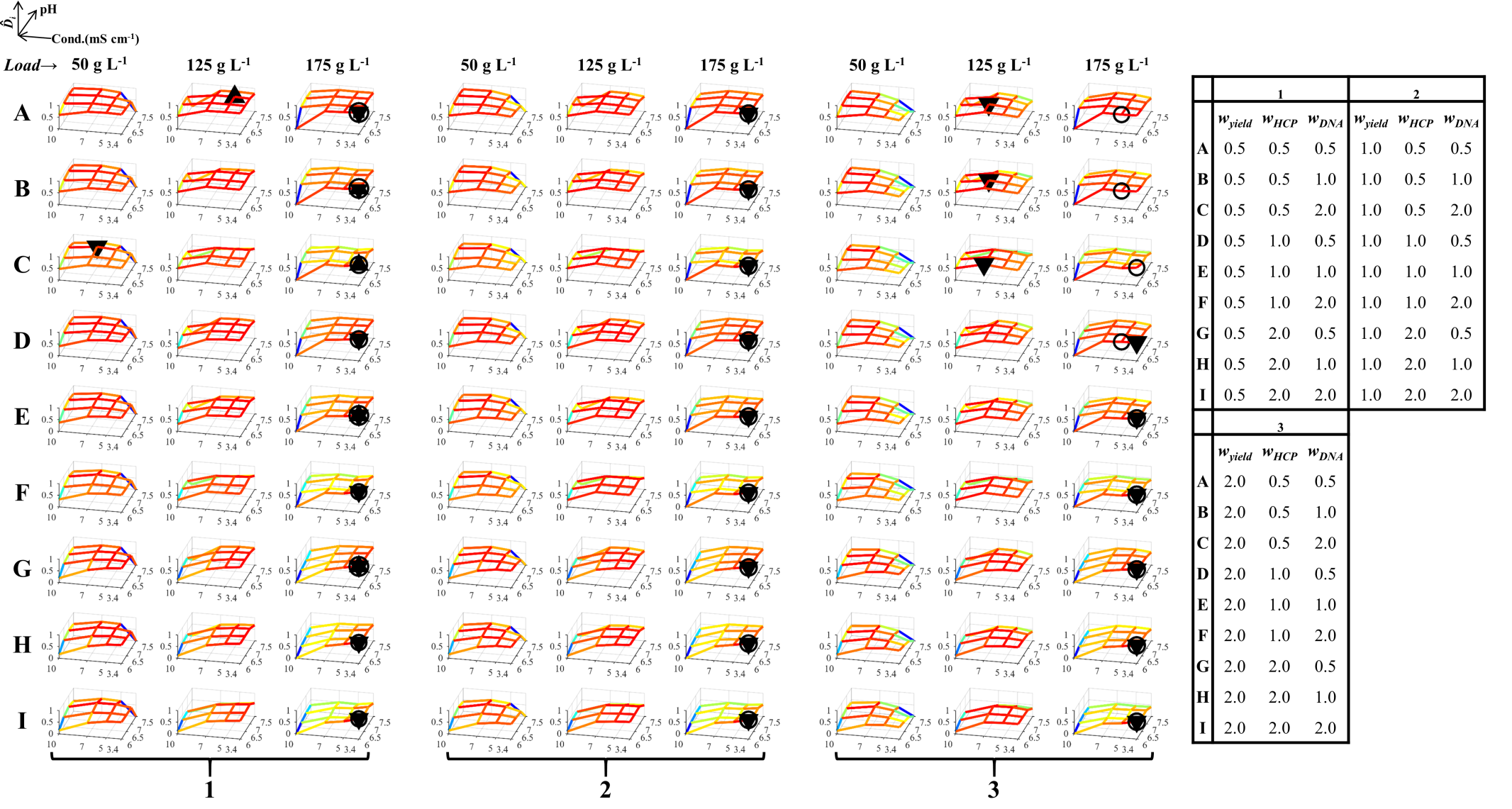


Figure S5: Mesh plot of predicted total desirability, $\hat{D}_{i}$, for Case Study 2 for the twenty seven sets of weights. Each row of plots, (A) – (I), corresponds to a different set of weights for the HCP and DNA content responses whereas the numbers at the bottom of the plots indicate different weights for the yield response. These are detailed in the table at the right hand side of the figure. (○) total desirability optima per set of weights based on the averaged raw measurements; (■) global scalar optimum (also Simplex–derived best condition); (▲) Simplex–derived local optimum; (▼) predicted optima per set of weights returned by the regression–based analysis approach. Blue color denotes low total desirability and red color denotes a high total desirability.


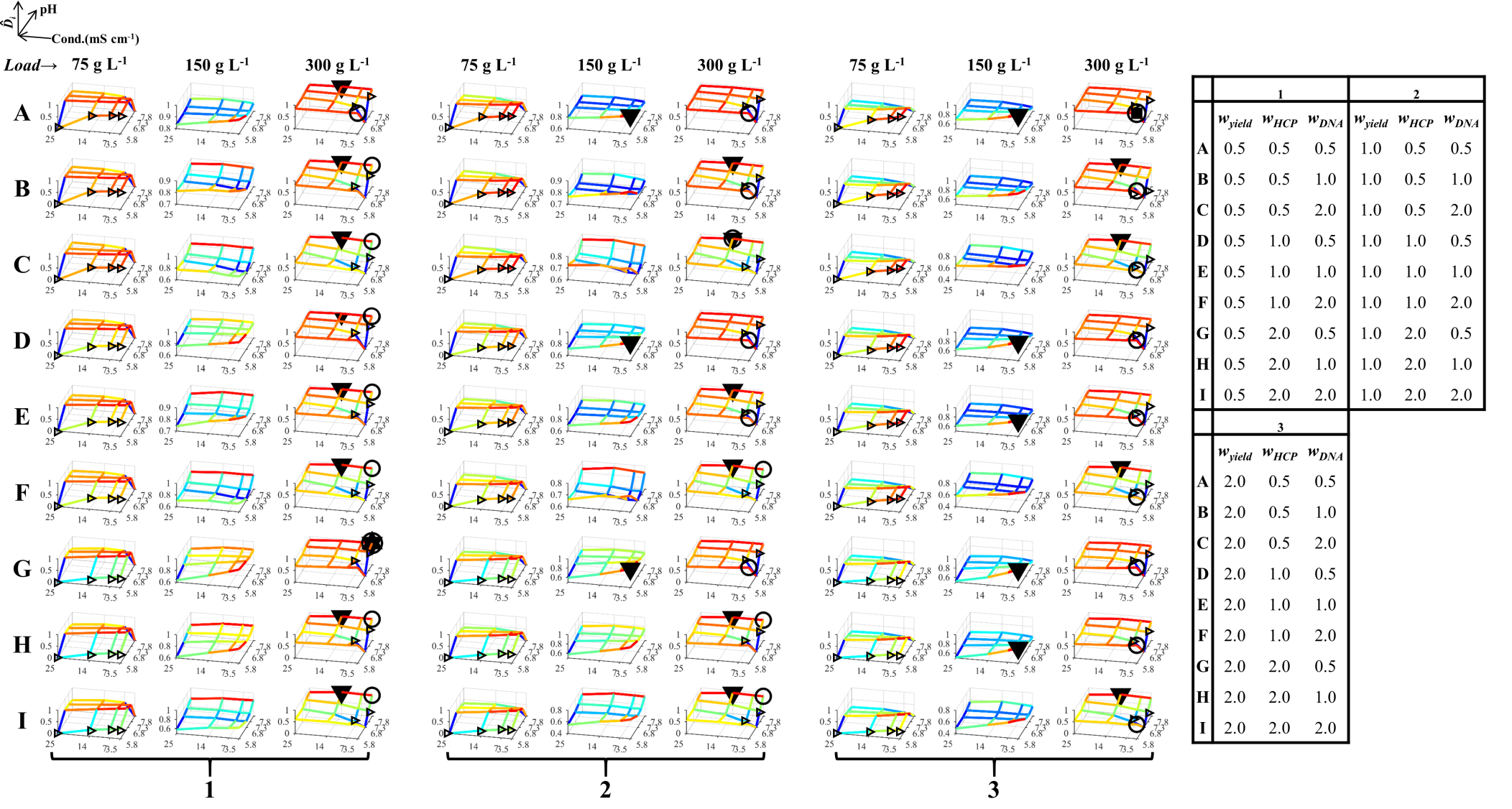


Figure S6: Mesh plot of predicted total desirability, $\hat{D}_{i}$, for Case Study 3 for the twenty seven sets of weights. Each row of plots, (A) – (I), corresponds to a different set of weights for the HCP and DNA content responses whereas the numbers at the bottom of the plots indicate different weights for the yield response. These are detailed in the table at the right hand side of the figure. (○) total desirability optima per set of weights based on the averaged raw measurements; (■) global scalar optimum (also Simplex–derived best condition); (▲) Simplex–derived local optimum; (▼) predicted optima per set of weights returned by the regression–based analysis approach; (▷) missing data points (replaced by a surrogate). Blue color denotes low total desirability and red color denotes a high total desirability.
